# Comparative efficacy of different exercise interventions for improving quality of life, depression, and anxiety in older adults with cancer: a systematic review and network meta-analysis

**DOI:** 10.1186/s12877-026-08022-4

**Published:** 2026-07-25

**Authors:** Wenhua Zhang, Xun Li, Dianbo Zhang, Jiecai Zheng

**Affiliations:** 1 College of Physical Education and Health, Guangxi Normal University, Guilin, 541006 Guangxi China; 2 College of Sport and Health, Shandong Sport University, Jinan, 250102 Shandong China; 3 School of Sport Communication and Information Technology, Shandong Sport University, Jinan, 250102 Shandong China; 4 School of Physical education, Shandong University, Jinan, 250061 Shandong China

**Keywords:** Older adults, Cancer, Exercise, Depression, Anxiety, Quality of life, NMA

## Abstract

**Objective:**

To systematically evaluate the effects of aerobic exercise (AE), resistance exercise (RE), mind-body exercise (MBE), and multimodal exercise (ME) on quality of life, depressive symptoms, and anxiety symptoms in older adults with cancer, and to compare the relative efficacy of different exercise types using network meta-analysis (NMA).

**Methods:**

A systematic search was conducted in PubMed, Cochrane Library, Embase, and Web of Science for randomized controlled trials published from database inception to April 5, 2026. Inclusion criteria were studies with participants having a mean age ≥ 65 years diagnosed with any type of cancer; interventions involving structured exercise training; and outcomes including quality of life, depression, or anxiety. Stata 17 was used for NMA, with standardized mean difference as the effect size. The surface under the cumulative ranking curve (SUCRA) was used to rank the efficacy of exercise types.

**Results:**

A total of 27 randomized controlled trials involving 2,573 older adults with cancer were included. SUCRA ranking showed that for improving quality of life, AE showed the highest-ranking probability (81.5%), followed by ME (76.3%), MBE (62.0%), and RE (53.1%). For improving depression, MBE showed the highest-ranking probability (98.6%), followed by ME (73.5%), AE (66.3%), and RE (31.2%). For improving anxiety, MBE also showed the highest-ranking probability (95.8%), followed by RE (75.7%), ME (62.0%), and AE (50.9%).

**Conclusion:**

The effects of different exercise types on psychological outcomes in older adults with cancer may vary. AE showed a higher-ranking probability for improving quality of life, while MBE demonstrated a higher-ranking probability for alleviating depression and anxiety. These findings should be interpreted cautiously considering the limitations of the evidence and may serve as a reference for clinical decision-making and future research.

**Supplementary Information:**

The online version contains supplementary material available at https://doi.org/10.1186/s12877-026-08022-4.

## Introduction

Population aging represents a global public health challenge. Cancer, being highly correlated with age, has an incidence and prevalence that continue to rise among the older adult population [1]. Global cancer statistics indicate approximately 19.96 million new cancer cases worldwide in 2022, a number projected to exceed 35 million by 2050, with older adults aged 60 years and above accounting for two-thirds of new cases [2]. This demographic shift renders the health management of older adults with cancer a critical issue in oncology.

Older adults with cancer face unique physiological and psychological challenges. Compared to younger individuals, this population often experiences diminished physiological reserve, increased comorbidity burden, cognitive decline, and weakened social support networks [3]. These factors predispose older adults with cancer to greater psychological distress during diagnosis, treatment, and recovery. Research indicates that approximately 30% to 35% of older adults with cancer have a diagnosable psychiatric disorder, and their suicide risk is 4.4 times higher than the general population [4]. Anxiety and depression, as the most common psychological symptoms, not only severely impair quality of life but may also influence disease prognosis by activating the hypothalamic-pituitary-adrenal axis and impairing immune surveillance [5].

Exercise intervention, as a safe, economical, and accessible non-pharmacological therapy, has gained extensive attention in the field of cancer rehabilitation [6]. Numerous randomized controlled trials (RCTs) have confirmed that regular exercise training can alleviate fatigue symptoms, improve physical function, and enhance mental health in cancer patients. The American College of Sports Medicine (ACSM) exercise guidelines for cancer survivors recommend that cancer patients engage in regular aerobic exercise (AE) and resistance exercise (RE) [7]. Exercise interventions can be classified by their physiological characteristics and training goals. AE involves rhythmic, continuous contraction of large muscle groups, relying on aerobic metabolism to improve cardiorespiratory fitness. Common forms include walking, running, stationary cycling, swimming, and treadmill/ergometer training. Prescription parameters typically include intensity (% maximum heart rate or RPE), frequency, duration, and total intervention period [6]. MBE integrates physical movements, breathing regulation, mindfulness, and meditation, emphasizing mind-body interaction and interoceptive awareness [8]. Common types include yoga, tai chi, and qigong, which are typically low-intensity and slow-paced, making them suitable for older adults with limited physical reserve [9]. RE enhances muscle strength, endurance, and mass by overcoming external resistance (e.g., free weights, elastic bands, machines). Common modalities include dumbbell/barbell, elastic band, and machine-based training [6]. Multimodal exercise (ME) refers to a comprehensive intervention combining two or more different exercise types, such as AE combined with RE, AE combined with MBE, RE combined with MBE, or a combination of all three, aiming to achieve multidimensional health benefits through the synergistic effects of multiple exercise modalities [10].

However, existing evidence primarily focuses on mixed-age cancer populations, with relatively limited research specifically targeting older adults with cancer. Furthermore, a systematic comparison of the relative efficacy of different exercise types (AE, RE, MBE, ME) on various psychological outcomes is lacking. Several recent systematic reviews have preliminarily explored the effects of exercise on psychological outcomes in older adults with cancer. Soong et al. [11] conducted a meta-analysis including 27 RCTs and found that exercise interventions significantly improved depression, anxiety, and quality of life in older adults with cancer. Wang et al. [12] performed an NMA comparing the effects of different exercise types on multiple symptoms in cancer survivors. Their results showed that AE was most effective for improving fatigue, RE was optimal for enhancing quality of life and relieving pain, MBE ranked first for improving anxiety, and multicomponent exercise was most effective for depression. Another NMA published in 2026 [13] similarly confirmed that AE was most effective for improving health-related quality of life, while MBE ranked highest for alleviating cancer-related fatigue.

However, the above-mentioned studies still have certain limitations. The meta-analysis by Soong et al. [11], although focusing on older adults with cancer, used traditional pairwise meta-analysis and could not provide indirect comparisons or efficacy ranking across different exercise types. The network meta-analyses (NMA) by Wang et al. [12] and Yu et al. [13] covered comparisons across multiple exercise types, but their included populations were mixed-age cancer survivors, and whether their conclusions can be directly generalized to older adults remains unclear. Older patients differ significantly from younger patients in terms of physiological reserve, comorbidity burden, and social support, and their tolerance, adherence, and response to exercise interventions may differ [3, 14]. Moreover, the above studies did not systematically compare the differential effects of different exercise types on quality of life, depression, and anxiety specifically in older adults.

To address these gaps, the present study differs from existing reviews in three aspects: population, outcomes, and classification. In terms of population, we focused on older adults with cancer to construct an NMA specifically for this population. In terms of outcomes, we simultaneously included quality of life, depression, and anxiety as three core psychological outcomes to examine the multidimensional effects of different exercise types. In terms of classification, we adopted a unified exercise classification standard (AE, RE, MBE, and ME) to systematically categorize interventions and enhance comparability across studies. This study aims to systematically search the relevant literature, compare the intervention effects of four exercise types on the above three psychological outcomes in older adults with cancer using NMA, and rank their efficacy using the surface under the cumulative ranking curve (SUCRA), thereby providing a reference for developing exercise prescriptions in this population.

## Materials and methods

### Protocol and registration

This systematic review strictly followed the Preferred Reporting Items for Systematic Reviews and Meta-Analyses (PRISMA) 2020 guidelines [15] and adhered to the PRISMA-NMA extension statement for standardized reporting of NMA-specific elements, including description of network geometry, transitivity assessment, inconsistency testing, and interpretation of ranking probabilities [15] (Appendix 1). The study protocol has been registered with the International Prospective Register of Systematic Reviews (PROSPERO) under the unique identifier CRD420261413377.

### Data sources and search strategy

A comprehensive systematic search was conducted in four electronic databases (PubMed, Cochrane Library, Embase, Web of Science) to identify RCTs investigating the effects of different exercise interventions on depression, anxiety, and quality of life in older adult cancer patients. To ensure complete coverage, the reference lists of included studies were also screened for additional literature. The search period covered from database inception to April 5, 2026. The search strategy was developed according to the PICOS framework (Population: older adults with cancer; Intervention: physical exercise; Comparison: any comparator; Outcomes: depression, anxiety, and quality of life measures; Study design: randomized controlled trials). Main search terms included: “Exercise”, “Physical Activity”, “Depression”, “Depressive Symptom”, “Anxiety”, “Anxiety Symptom”, “Quality of Life”, “Cancer”, “Older Adults”, “Geriatrics”, etc. The detailed search strategy is presented in Appendix 2.

### Inclusion and exclusion criteria

#### Inclusion criteria

The study selection criteria were based on the PICOS framework. Studies meeting all the following criteria were included:

1. Study design: English-language, peer-reviewed RCT.
2. Study population: Patients aged ≥ 65 years or with a study sample mean age ≥ 65 years, diagnosed with any type of cancer (regardless of comorbidities). For studies reporting the overall sample mean age rather than individual-level age, they were included if the mean age met the ≥ 65-year criterion.
3. Interventions: Studies assessing the association between exercise intervention and psychological outcomes were included. Exercise intervention was defined as activity exceeding daily physical function, including AE (e.g., walking, running/jogging, stationary cycling, swimming, and treadmill/ergometer training), RE (e.g., elastic bands, dumbbells/barbells, and machine-based training), ME (combining two or more exercise modalities, such as AE + RE, AE + MBE, etc.), and MBE (e.g., Qigong, Yoga, Tai Chi). The above exercise classification was based on the following principles: (i) exercise physiological characteristics—distinguished by the primary energy system, muscle contraction mode, and degree of mind-body integration [6]; (ii) preset intervention goals—based on the primary training purpose explicitly specified by the intervention designers; and (iii) operational definitions—AE, RE, and MBE each have well-established physiological characteristics and clinical evidence as independent exercise modalities. When a primary study included two or more exercise modalities simultaneously, it was uniformly classified under the ME node to examine the synergistic effects of combined interventions.
4. Comparator: The control group received usual care, waitlist control, or active control. Specifically: (i) Usual care refers to patients receiving standardized oncological treatment and routine follow-up at their medical institution, without any additional study intervention; (ii) Waitlist control refers to patients who do not receive the intervention during the trial period but are offered the same protocol as the intervention group after the study is completed; and (iii) Active control refers to the control group receiving some alternative activity (e.g., stretching exercises or conventional physical activity) to control for non-specific effects (such as additional attention, social interaction, or activity exposure), provided that this activity does not fall within the four types of structured exercise interventions defined in this study. If the active control included conventional exercise or structured activity, this was recorded and considered in the interpretation of the results.
5. Outcomes: Depression, anxiety, and health-related quality of life (HRQOL), with at least one of these three psychological outcomes analyzed.
6. Data completeness: Sufficient original or extractable data (means, standard deviations, sample sizes) to calculate effect sizes (e.g., standardized mean difference).

#### Exclusion criteria

Studies meeting any of the following criteria were excluded:

1. Study design: Observational studies (e.g., cross-sectional, case-control, cohort studies).
2. Study population: Patient groups with mean age < 65 years, pre-cancerous lesions (e.g., early forms of ductal carcinoma in situ, or colonic adenomas), benign tumors, patients with non-cancer chronic diseases (e.g., heart disease, diabetes, arthritis alone), and healthy populations.
3. Intervention: Studies with insufficient protocol details (e.g., unquantified intensity/duration) or combined interventions where the effect of exercise could not be isolated (e.g., exercise combined with dietary supplements), studies involving only educational interventions, pharmacological interventions, or surgical interventions.
4. Study type: Qualitative studies, reviews, theses, or conference papers.
5. Incomplete data: Missing key outcome data or data not extractable (e.g., descriptive statistics only without numerical values).
6. Ethical issues: Violations of ethical standards (e.g., lack of informed consent, inappropriate risk-benefit ratio).

### Data extraction

Retrieved records were imported into Endnote 20 software, and duplicates were removed. Subsequently, two reviewers independently screened the literature and extracted information according to the predefined inclusion and exclusion criteria. Disagreements were resolved through discussion with a third reviewer. Extracted characteristics of included studies included: author and year, country of publication, study design, sample size, age, cancer type, outcome measures, assessment tools, exercise type, exercise frequency, exercise duration, and control group activities. For studies reporting multiple follow-up time points, we preferentially extracted data from the time point closest to the end of the intervention as the primary analysis time point to assess the short-term direct effects of the exercise intervention.

### Quality assessment

The risk of bias of the included studies was assessed using the Cochrane Risk of Bias tool version 2.0 (RoB 2). Two reviewers independently evaluated five domains for each trial: the randomization process, deviations from intended interventions, missing outcome data, measurement of the outcome, and selection of the reported result. Each domain was judged as “low risk,” “some concerns,” or “high risk” based on the pre-specified signaling questions and algorithms in the RoB 2 official guidance. Based on the domain-level judgments, an overall risk-of-bias judgment was derived according to the RoB 2 algorithm: if any domain was rated as “high risk,” the overall judgment was “high risk”; if any domain was rated as “some concerns” and no domain was rated as “high risk,” the overall judgment was “some concerns”; only when all domains were rated as “low risk” was the overall judgment “low risk.” Disagreements between the two reviewers were resolved through discussion with a third reviewer to reach consensus.

### Statistical analysis

RStudio and Stata 17 software was used to perform traditional pairwise meta-analysis and NMA on the included outcomes. Because different studies used different assessment scales, continuous data were analyzed using the standardized mean difference (SMD) with 95% confidence intervals (CI) as the effect size. To ensure consistency in the direction of all effect sizes, the following standardization was performed after calculating the SMD: for quality of life scales, where higher scores indicate better quality of life, the original SMD direction was retained (positive values favoring the intervention); for depression and anxiety scales, where higher scores indicate more severe symptoms, the calculated SMD was multiplied by − 1 so that negative SMD values favor the intervention. Statistical inference was conducted through heterogeneity testing and pooling of effect sizes. Heterogeneity was assessed using the I² statistic [16]. The effect model was selected based on the degree of heterogeneity: a fixed-effect model was used when I² ≤ 50%, and a random-effects model was used when I² > 50% [17]. The fixed-effect model assumes that all studies estimate the same true effect size and is appropriate when between-study heterogeneity is low; the random-effects model allows the true effect size to vary across studies and is appropriate when heterogeneity is high [17]. When heterogeneity is low, the fixed-effect model provides more precise effect estimates, and the results of the two models tend to converge when heterogeneity is small [18]. Network evidence plots showing direct comparisons between different exercise interventions were generated. Forest plots of pairwise comparisons were used to present the NMA results. The SUCRA was used to rank exercise types by efficacy probability, with higher SUCRA values indicating a greater probability of ranking higher for a given outcome. SUCRA values reflect the probability that an intervention ranks highest among all interventions in a specific network, rather than being a deterministic measure of absolute efficacy. SUCRA results should be interpreted in conjunction with the width of confidence intervals for effect sizes, the composition of direct versus indirect evidence, and clinical heterogeneity.

For multi-arm RCTs, this study retained their full original structure in the NMA. When using the network command suite in Stata 17, the command automatically accounted for the correlations between multiple comparisons within the same study by constructing a block-diagonal variance-covariance matrix, thereby ensuring the accuracy of effect size estimates and avoiding unit-of-analysis error [19].

### Consistency assessment

Global consistency of the entire network was assessed using a global consistency model. A *P* value < 0.05 was considered to indicate significant global inconsistency. Local inconsistency was assessed using the node-splitting method. A statistically significant difference between direct and indirect evidence (*P* < 0.05) was considered to indicate local inconsistency.

### subgroup analysis

To further assess the potential impact of clinical heterogeneity and evaluate the consistency of exercise intervention effects across different clinical characteristics, this study pre-planned subgroup analyses. Subgroup analyses were performed according to risk of bias, intervention duration (< 12 weeks vs. ≥12 weeks), comparator type (usual care/waitlist vs. active control), cancer type, metastatic status (metastatic vs. non-metastatic), and disease phase (active treatment vs. survivorship). Due to the limited number of studies in some subgroups, the results of subgroup analyses are presented as exploratory analyses only.

### Publication bias

If the NMA included ≥ 10 studies, Egger’s test was used to detect publication bias. Publication bias risk was assessed using two methods: visual inspection of funnel plots generated with Stata software for asymmetry [20], and Egger’s test [21].

### Sensitivity analysis

Sensitivity analysis was conducted using a leave-one-out approach, sequentially excluding each individual study, to assess the robustness of the results.

### Transitivity assessment

To evaluate the transitivity assumption of this NMA, we compared the distribution of key effect modifiers across intervention nodes, including age, sample size, intervention duration, exercise frequency, cancer type, treatment phase, baseline psychological status, exercise intensity, supervision, metastatic status, survivorship phase, comparator type, and outcome measurement instruments. If the distribution of the above characteristics was generally balanced across nodes, the transitivity assumption was satisfied.

### Certainty of evidence assessment

The certainty of evidence for each comparison was assessed using the CINeMA (Confidence in Network Meta-Analysis) online tool. The assessment was based on six domains: within-study bias, reporting bias, indirectness, imprecision, heterogeneity, and incoherence. Each domain was automatically rated according to the pre-specified algorithms in CINeMA, and the combined results yielded the certainty of evidence grades: high, moderate, low, or very low.

## Results

### Literature search results

**Fig. 1:**
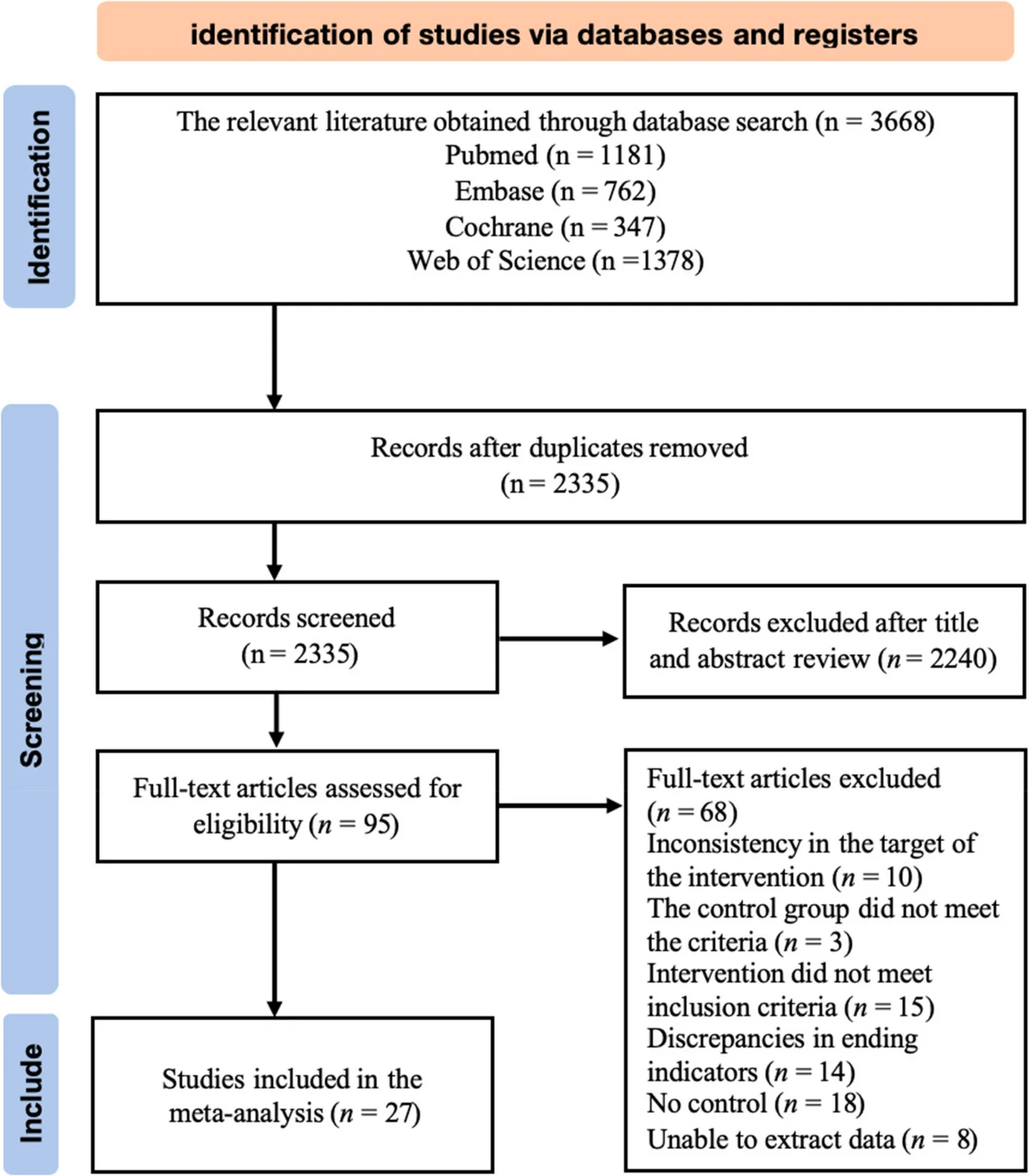
PRISMA literature screening flow diagram

A total of 3,668 records were retrieved from PubMed, Embase, Web of Science, and Cochrane Library. After screening titles, abstracts, and full texts according to the inclusion and exclusion criteria, 27 studies were ultimately included in the meta-analysis. The screening flow diagram is shown in Fig. 1.

### Characteristics of included studies

A total of 27 studies published between 2003 and 2024 were included, with a total of 2,573 participants (1,375 in the exercise groups and 1,198 in the control groups). Regarding country of publication: United States (*n* = 5), Australia (*n* = 4), Canada (*n* = 3), United Kingdom (*n* = 2), Norway (*n* = 2), China (*n* = 2), Denmark (*n* = 1), Portugal (*n* = 1), Japan (*n* = 1), Turkey (*n* = 1), Germany (*n* = 1), Netherlands (*n* = 1), Belgium (*n* = 1), Iran (*n* = 1), and Sweden (*n* = 1). Twenty-five RCTs examined quality of life, 15 specifically examined depression, and 11 specifically examined anxiety. Exercise intervention durations ranged from 1 week to 12 months. Exercise types were classified as follows: AE (*n* = 7; including walking, stationary cycling, treadmill training, etc.); RE (*n* = 8; including elastic bands, dumbbells/barbells, and machine-based training); MBE (*n* = 4; including yoga, qigong, and tai chi); ME (*n* = 12). The study characteristics are detailed in Tables 1 and 2.

**Table 1:** Basic characteristics of included studies

Study	Country	Mean/Age, y	Sample size, *n*	Cancer type	Treatment phase	Metastatic status	Survivorship phase	Supervision	Outcome measures	Assessment tool
Segal [22] (2003)	Canada	E: 68.2 C: 67.7	E: 82 C: 73	Prostate cancer	During hormone therapy	Non-metastatic	Active treatment phase	Fully supervised	Quality of life	FACT-P
Monga [23] (2007)	USA	E: 68.0 C: 70.6	E: 11 C: 10	Head and neck cancer	During radiotherapy	Non-metastatic	Active treatment phase	Fully supervised	Quality of life, depression	FACT-P, BDI
Segal [24] (2009)	Canada	E1: 66.4 E2: 66.2 C: 65.3	E1: 40 E2: 40 C: 41	Prostate cancer	During radiotherapy (± hormone therapy)	Non-metastatic	Active treatment phase	Fully supervised	Quality of life	FACT-G
Culos-Reed [25] (2010)	Canada	E: 67.2 C: 68.0	E: 53 C: 47	Prostate cancer	During hormone therapy	Non-metastatic	Active treatment phase	Partially supervised	Quality of life, depression	EORTC QLQ-C30, CES-D
Bourke [26] (2011)	UK	E: 71.3 C: 72.2	E: 25 C: 25	Prostate cancer	During hormone therapy	Non-metastatic	Active treatment phase	Partially supervised	Quality of life	FACT-G
Cormie [27] (2013)	Australia	E: 73.1 C: 71.2	E: 10 C: 10	Prostate cancer	During hormone therapy	Metastatic (bone metastases)	Active treatment phase	Fully supervised	Quality of life, depression, anxiety	SF-36, BSI-18
Arbane [28] (2014)	UK	E: 67 C: 68	E: 64 C: 67	Lung cancer	Postoperative	Non-metastatic	Survivorship	Partially supervised	Quality of life	SF-36
Edvardsen [29] (2014)	Norway	65.2	E: 64 C: 67	Lung cancer	Postoperative	Non-metastatic	Survivorship	Partially supervised	Quality of life	SF-36
Galvão [30] (2014)	Australia	E: 71.9 C: 71.5	E: 50 C: 50	Prostate cancer	After radiotherapy and hormone therapy	Non-metastatic	Survivorship	Partially supervised	Quality of life	SF-36
Campo [31] (2014)	USA	73.8	E: 20 C: 20	Prostate cancer	Post-surgery/radiotherapy/hormone therapy	Non-metastatic	Survivorship	Fully supervised	Depression, anxiety	BSI-18
Miki [32] (2014)	Japan	E: 72.97 C: 75.45	E: 38 C: 40	Breast or prostate cancer	During chemotherapy/hormone therapy/radiotherapy	Non-metastatic	Active treatment phase	Fully supervised	Quality of life	FACT-G
Cormie [33] (2015)	Australia	E: 69.6 C: 67.1	E: 32 C: 31	Prostate cancer	Early phase of hormone therapy	Non-metastatic	Active treatment phase	Fully supervised	Quality of life, depression, anxiety	SF-36, BSI-18
Nilsen [34] (2015)	Norway	66	E: 28 C: 30	Prostate cancer	During hormone therapy	Non-metastatic	Active treatment phase	Fully supervised	Quality of life	EORTC QLQ-C30
Yagli [35] (2015)	Turkey	E: 68.58 C: 68.88	E: 10 C: 10	Breast cancer	After chemotherapy	Non-metastatic	Survivorship	Fully supervised	Quality of life, depression	NHP, BDI
Cramer [36] (2016)	Germany	68.26	E: 27 C: 27	Colorectal cancer	Postoperative	Non-metastatic	Survivorship	Fully supervised	Quality of life, depression, anxiety	FACT-C, HADS
Winters-Stone [37] (2016)	USA	E: 70.6 C: 72.9	E: 32 C: 32	Prostate cancer	Post-primary treatment (surgery/radiotherapy)	Non-metastatic	Survivorship	Fully supervised	Quality of life	SF-36
Lai [38] (2017)	China	E: 72.5 C: 71.6	E: 30 C: 30	Lung cancer	Preoperative	Non-metastatic	Active treatment phase	Fully supervised	Quality of life	EORTC QLQ-C30
Cavalheri [39] (2017)	Australia	E: 66 C: 68	E: 30 C: 30	Lung cancer	Postoperative	Non-metastatic	Survivorship	Fully supervised	Quality of life, depression, anxiety	SF-36, HADS
Golsteijn [40] (2018)	Netherlands	E: 66.55 C: 66.38	E: 249 C: 229	Prostate and colorectal cancer	During or after treatment	Non-metastatic	Active treatment phase	Unsupervised	Quality of life, depression, anxiety	EORTC QLQ-C30, HADS
Loh [41] (2019)	USA	E: 67.7 C: 65.5	E: 130 C: 122	Breast cancer	During chemotherapy	Non-metastatic	Active treatment phase	Unsupervised	Quality of life, depression, anxiety	FACT-G, POMS, STAI
Piraux [42] (2021)	Belgium	69.1	E1: 26 E2: 26 C: 26	Prostate cancer	During radiotherapy	Non-metastatic	Active treatment phase	Fully supervised	Quality of life, depression	FACT-G, CES-D
Mardani [43] (2021)	Iran	E: 69.40 C: 70.39	E: 40 C: 40	Prostate cancer	Post-radiotherapy/hormone therapy	Non-metastatic	Survivorship	Partially supervised	Quality of life	EORTC QLQ-C30
Cheng [44] (2021)	China	66.3	E1: 30 E2: 30 C: 30	Lung, stomach, or breast cancer	During chemotherapy/radiotherapy	Non-metastatic	Active treatment phase	Fully supervised	Depression, anxiety	PHQ-9, STAI
Mikkelsen [45] (2022)	Denmark	71.7	E: 41 C: 43	Pancreatic, biliary, or NSCLC	During first-line palliative therapy	Metastatic	Survivorship	Fully supervised	Quality of life, depression, anxiety	EORTC QLQ-C30, HADS
Capela [46] (2023)	Portugal	E: 72.8 C: 70.7	E: 16 C: 15	Prostate cancer	During hormone therapy	Non-metastatic	Active treatment phase	Fully supervised	Quality of life	EORTC QLQ-C30
Langlais [47] (2023)	USA	69.3	E1: 8 E2: 8 C: 10	Prostate cancer	Advanced/palliative phase	Metastatic	Active treatment phase	Fully supervised	Quality of life, depression, anxiety	FACT-G, CES-D, STAI
Porserud [48] (2024)	Sweden	E: 71.3 C: 71.3	E: 47 C: 43	Bladder cancer	Postoperative	Non-metastatic	Survivorship	Fully supervised	Quality of life, depression, anxiety	EORTC QLQ-C30, HADS

*E* Exercise group, *C* Control group, *FACT-P* Functional Assessment of Cancer Therapy-Prostate, *FACT-G* Functional Assessment of Cancer Therapy-General, *EORTC QLQ-C30* European Organisation for Research and Treatment of Cancer Quality of Life Questionnaire Core 30, *SF-36* 36-Item Short Form Health Survey, *FACT-C* Functional Assessment of Cancer Therapy-Colorectal, *NHP* Nottingham Health Profile, *BDI* Beck Depression Inventory, *CES-D* Center for Epidemiologic Studies Depression Scale, *HADS* Hospital Anxiety and Depression Scale, *PHQ-9* Patient Health Questionnaire-9, *STAI* State-Trait Anxiety Inventory, *BSI-18* Brief Symptom Inventory-18, *POMS* Profile of Mood States, *NSCLC* Non-small cell lung cancer

**Table 2:** Basic exercise characteristics of the included studies

Study	Exercise type	Comparator type	Exercise duration	Exercise frequency	Session duration	Exercise intensity
Segal [22] (2003)	RE (leg extension, chest press, lat pulldown, etc.)	Waitlist control	12 weeks	3 times/week	NR	60%~70% 1RM
Monga [23] (2007)	AE (treadmill walking)	Usual care	8 weeks	3 times/week	30 ~ 40 min	65% HRR
Segal [24] (2009)	E1: AE (stationary cycling/treadmill/elliptical)	Usual care	24 weeks	3 times/week	15→45 min	50%~75% VO₂peak
Segal [24] (2009)	E2: RE (leg extension, leg curl, chest fly, etc.)	Usual care	24 weeks	3 times/week	NR	60%~70% 1RM
Culos-Reed [25] (2010)	ME (AE + RE)	Waitlist control	16 weeks	3 ~ 5 times/week	NR	Moderate intensity
Bourke [26] (2011)	ME (AE + RE)	Usual care	12 weeks	2 ~ 3 times/week	30 min (AE**)**	Moderate intensity
Cormie [27] (2013)	RE (8 machine-based exercises)	Usual care	12 weeks	2 times/week	60 min	12 − 8 RM
Arbane [28] (2014)	ME (AE + RE)	Usual care	4 weeks	NR	NR	Moderate intensity
Edvardsen [29] (2014)	ME (chest press, leg press, etc.+ cycling/walking)	Usual care	20 weeks	3 times/week	60 min	AE: 80%–95% HRmax; RE: 6–12 RM
Galvão [30] (2014)	ME (AE + RE)	Usual care	12 months	2 ~ 4 times/week	NR	AE: 70%–85% HRmax; RE: 6–12 RM
Campo [31] (2014)	MBE (Qigong)	Active control (stretching exercise)	12 weeks	2 times/week	60 min	Low intensity (Borg 4.3/10)
Miki [32] (2014)	AE (stationary cycling)	Usual care	4 weeks	1 times/week	5 min	20 W, 80 rpm
Cormie [33] (2015)	ME (abdominal breathing + expiratory exercise + AE)	Usual care	12 weeks	2 times/week	60 min	AE: 70%–85% HRmax; RE: 6–12 RM
Nilsen [34] (2015)	RE (Smith machine half squat, leg press, chest press, etc.)	Usual care	16 weeks	3 times/week	NR	6 ~ 10 RM
Yagli [35] (2015)	MBE (Yoga)	Active control (conventional exercise)	8 weeks	2 times/week	60 min	NR
Cramer [36] (2016)	MBE (Yoga)	Waitlist control	10 weeks	1 times/week	90 min	NR
Winters-Stone [37] (2016)	RE (elastic bands + free weights + weighted vest)	Usual care	6 months	2 times/week	60 min	4%–15% of body weight; 8–15 RM
Lai [38] (2017)	ME (walking/treadmill/cycling + step-ups, hand weights)	Usual care	1 week	Daily	NR	Progressive loading
Cavalheri [39] (2017)	ME (AE + RE)	Usual care	8 weeks	3 times/week	60 min	AE: 80% of 6MWT speed; RE: 1.5–2 kg
Golsteijn [40] (2018)	AE (walking)	Waitlist control	3 months	NR	NR	Moderate intensity
Loh [41] (2019)	ME (AE + RE)	Usual care	6 weeks	Daily	NR	AE: 5%–20% progressive increase; RE: RPE 3–5/10
Piraux [42] (2021)	E1: AE (stationary cycling)	Usual care	5–8 weeks	3 times/week	26 ~ 40 min	≥ 85% HRmax
Piraux [42] (2021)	E2: RE (elastic bands/dumbbells/body weight)	Usual care	5–8 weeks	3 times/week	30 min	RPE 4 ~ 6/10
Mardani [43] (2021)	ME (AE + RE+ flexibility exercises + pelvic floor)	Usual care	12 weeks	4 times/week	NR	AE: Borg 11–13; RE: NR
Cheng [44] (2021)	E1: MBE (Tai Chi)	Usual care	12 weeks	3 times/week	40 min	NR
Cheng [44] (2021)	E2: RE (standing row, bench press, dumbbell press, etc.)	Usual care	12 weeks	3 times/week	NR	30%~60% 1RM
Mikkelsen [45] (2022)	ME (RE + walking program + protein supplement)	Usual care	12 weeks	2 ~ 3 times/week	60 min	RE: 10 ~ 15 RM
Capela [46] (2023)	AE (walking football)	Usual care	16 weeks	3 times/week	90 min	72.8% HRmax
Langlais [47] (2023)	E1: AE (stationary cycling)	Usual care	12 weeks	3 times/week	NR	High-intensity interval training
Langlais [47] (2023)	E2: RE (8 machine-based exercises)	Usual care	12 weeks	3 times/week	NR	NR
Porserud [48] (2024)	ME (AE+ strengthening + pelvic floor)	Active control (home-based exercise)	12 weeks	2 times/week	NR	Moderate intensity

*E* Exercise group, *C* Control group, *AE* Aerobic exercise, *MBE* Mind-body exercise, *ME* Multimodal exercise, *RE* Resistance exercise, *1RM* one-repetition maximum, *HRR* Heart rate reserve, *HRmax* maximum heart rate, *VO*_2_*peak* peak oxygen consumptio, *RPE* Rating of perceived exertion, *W* watt, *rpm* revolutions per minute, *RM* Repetition maximum, *6MWT* 6-minute walk test, *HIIT* High-intensity interval training, *NR* Not reported

### Risk of bias

**Fig. 2:**
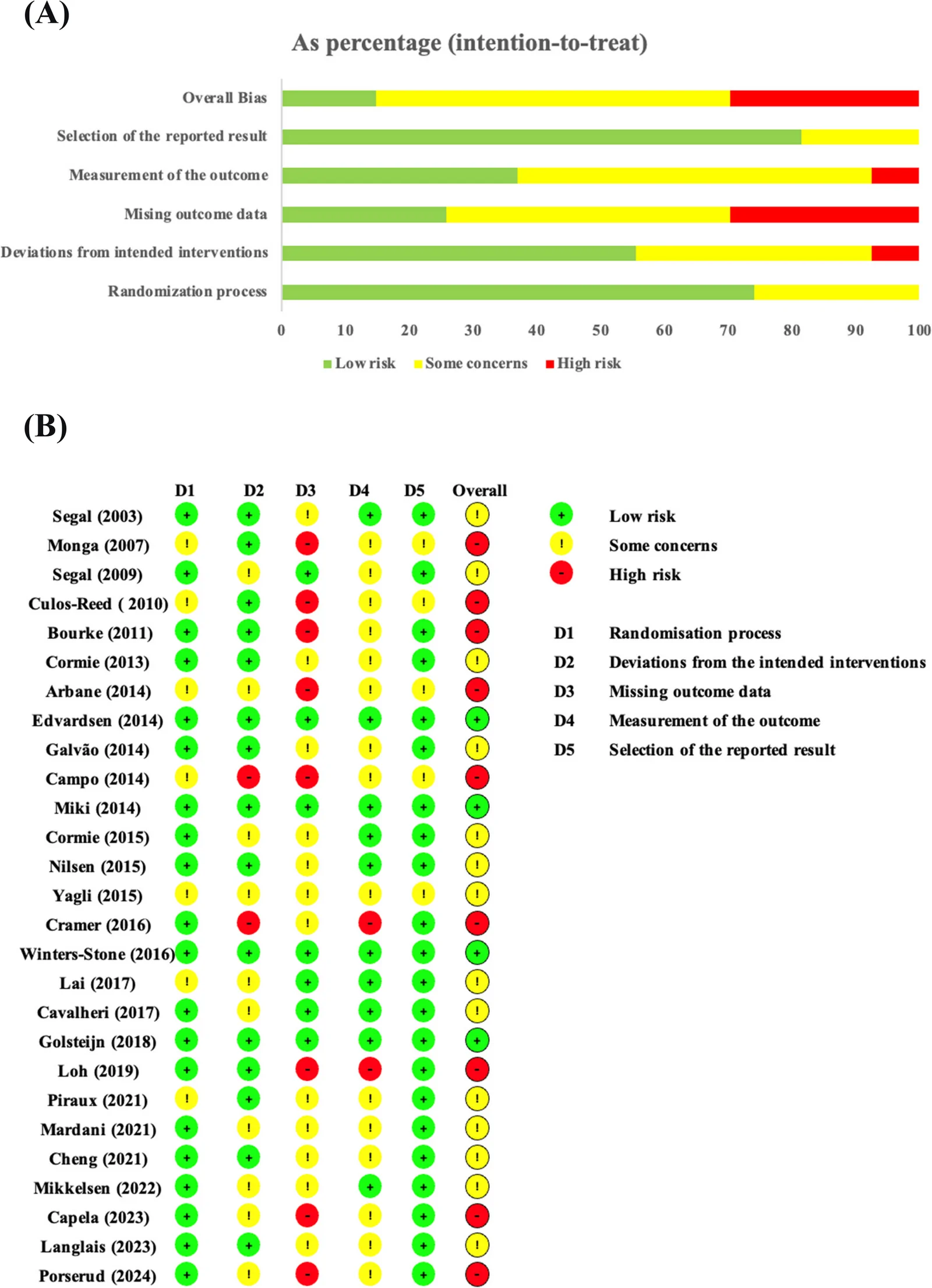
Risk-of-bias summary for the 27 randomized controlled trials assessed with the Cochrane RoB 2 tool. **A** Proportion of trials assigned low, some-concern, or high risk for each domain, expressed as percentages. **B** Individual risk-of-bias judgments for all six domains across each of the 27 included trials

Among the 27 included RCTs, 24 reported specific methods for random sequence generation, primarily using computer-generated random numbers or random number tables; 13 reported specific methods for allocation concealment (e.g., sealed opaque envelopes); 16 reported blinding of outcome assessors, but participants could not be blinded to the exercise interventions due to the nature of the intervention. All 27 studies reported the number of dropouts and losses to follow-up. Regarding analytical methods, 2 studies used per-protocol (PP) analysis, 17 used intention-to-treat (ITT) analysis, and 8 reported both PP and ITT analyses. Twenty-three studies reported clinical trial registration numbers. The results of the risk-of-bias assessment for each domain are shown in Fig. 2.

### Network evidence plot

**Fig. 3:**
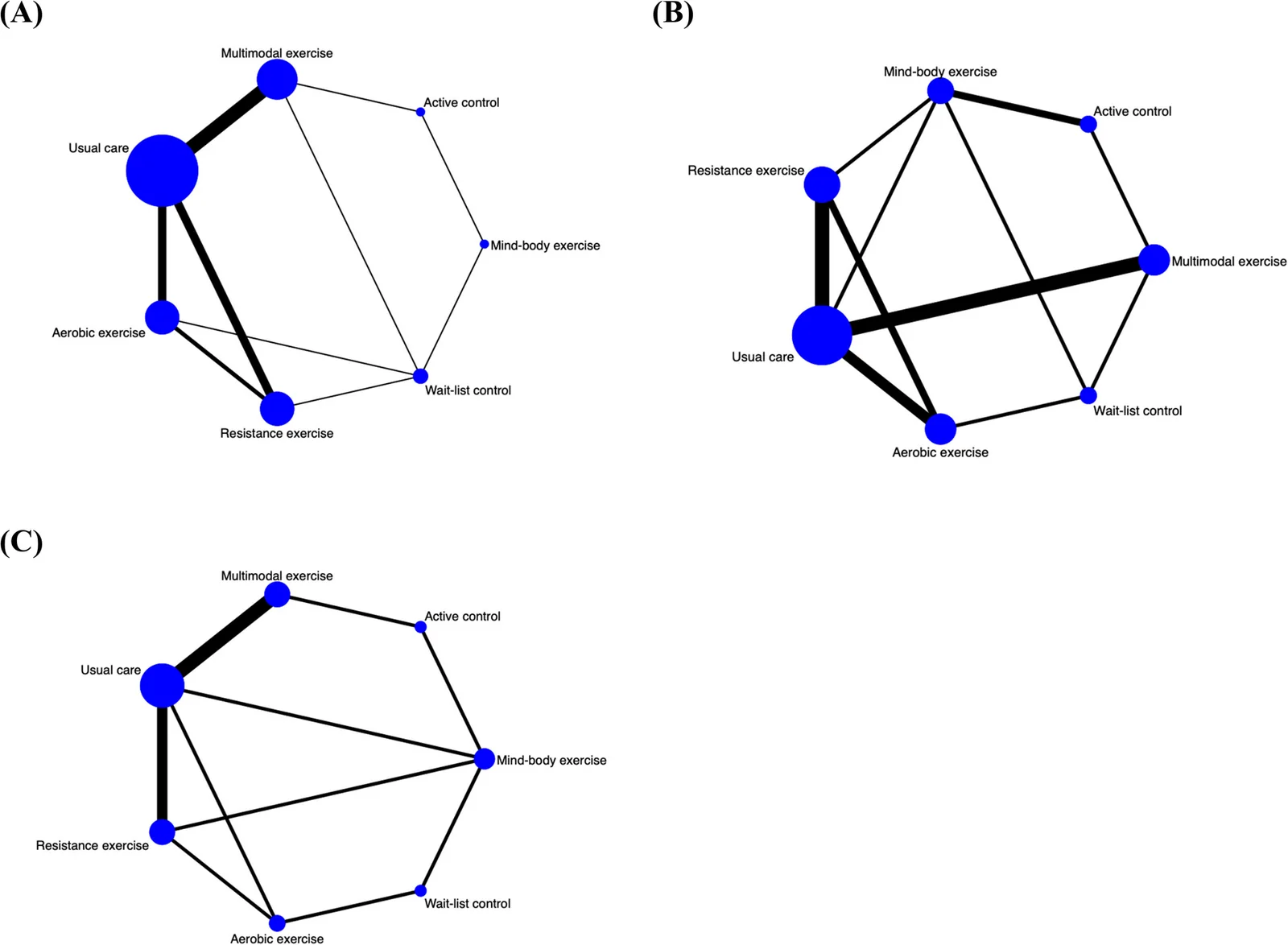
Network evidence plots. **A** Quality of life, (**B**) Depression, (**C**) Anxiety

Based on traditional meta-analysis, the NMA included 27 studies with 2,573 participants. In the network plots (Fig. 3), nodes represent exercise types, node sizes represent the sample size, and lines between nodes indicate the presence of direct comparisons between two interventions. Thicker lines indicate a larger number of studies. The absence of a line between two exercise types indicates no direct comparison, allowing indirect comparisons via NMA.

In the quality-of-life network, direct comparisons were most frequent for AE versus usual care (*k* = 6) and ME versus usual care (*k* = 7). In the depression network, direct comparisons were concentrated for MBE versus usual care (*k* = 3) and ME versus usual care (*k* = 4). The anxiety network was relatively sparse, with ME versus usual care (*k* = 4) as the primary source of evidence. All three networks contained indirect comparisons (e.g., MBE versus RE lacked direct evidence), and results based on indirect evidence should be interpreted with caution.

### NMA of outcomes

In the 27 included RCTs, usual care was the most common comparator, while active control was used in only a few studies. Notably, the content of active control varied across studies—some used stretching or conventional physical activity, which may have physiological and psychological effects that could narrow the between-group differences. Therefore, comparisons involving active control may yield conservative estimates and should be interpreted with caution. In this study, SMD was standardized so that positive values favor the intervention for quality of life, while negative values favor the intervention for depression and anxiety.

#### Quality of life

**Fig. 4:**
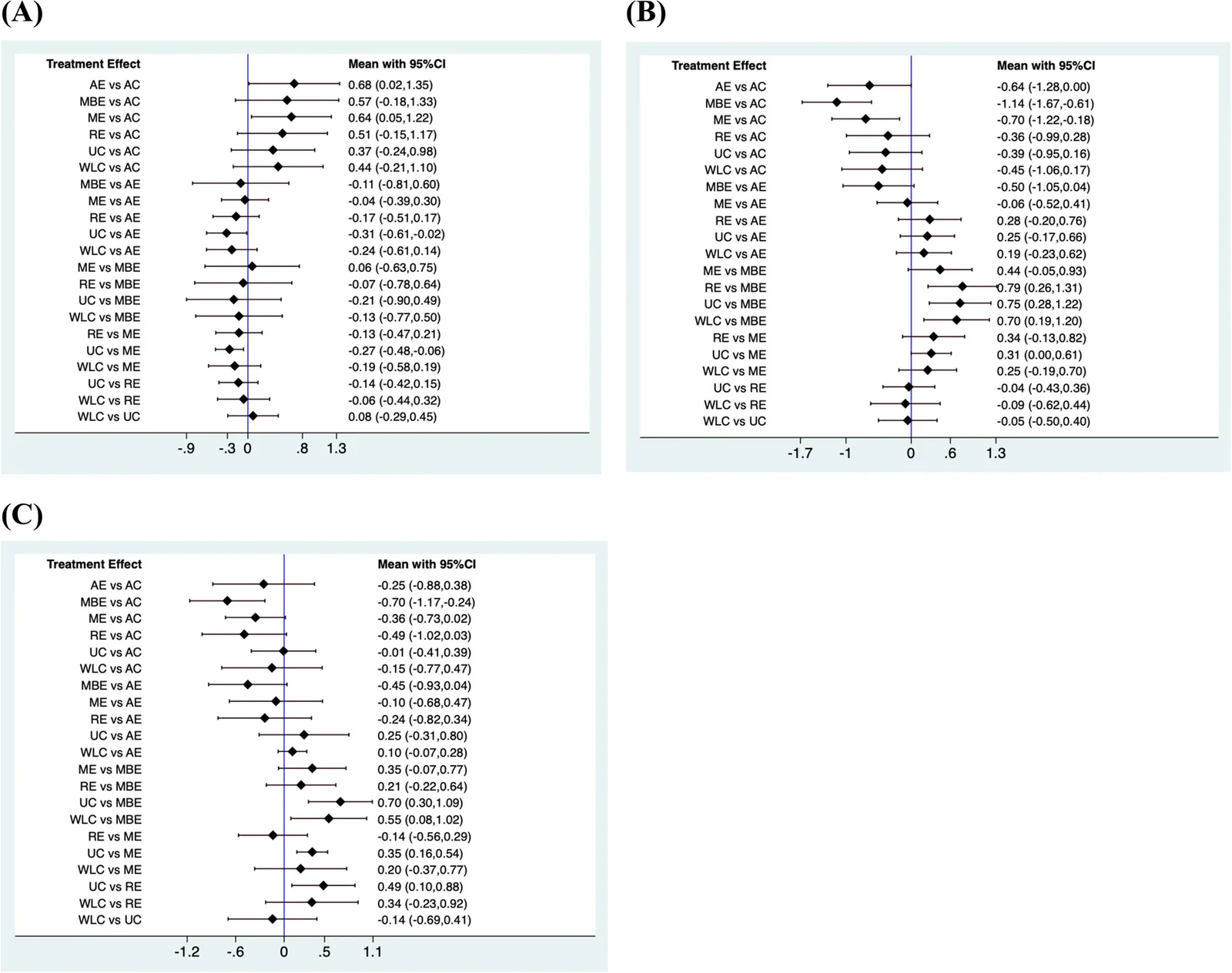
NMA forest plots. (**A**) Quality of life, (**B**) Depression, (**C**) Anxiety. AE, aerobic exercise; MBE, mind-body exercise; ME, multimodal exercise; RE, resistance exercise; WLC, waitlist control; UC, usual care; AC, active control

Twenty-five studies examined the effects of different exercise types on improving quality of life in older adults with cancer, involving 7 interventions (4 exercise types and 3 control types). NMA showed statistically significant results as follows: AE (SMD = 0.31, 95% CI: 0.02 to 0.61) and ME (SMD = 0.27, 95% CI: 0.06 to 0.48) were both significantly superior to usual care; AE (SMD = 0.68, 95% CI: 0.02 to 1.35) and ME (SMD = 0.64, 95% CI: 0.05 to 1.22) were also significantly superior to active control. Beyond these significant results, most other comparisons showed positive trends, but their 95% CIs crossed zero, indicating insufficient evidence for statistical significance (Fig. 4A, Figure S3.1).

**Fig. 5:**
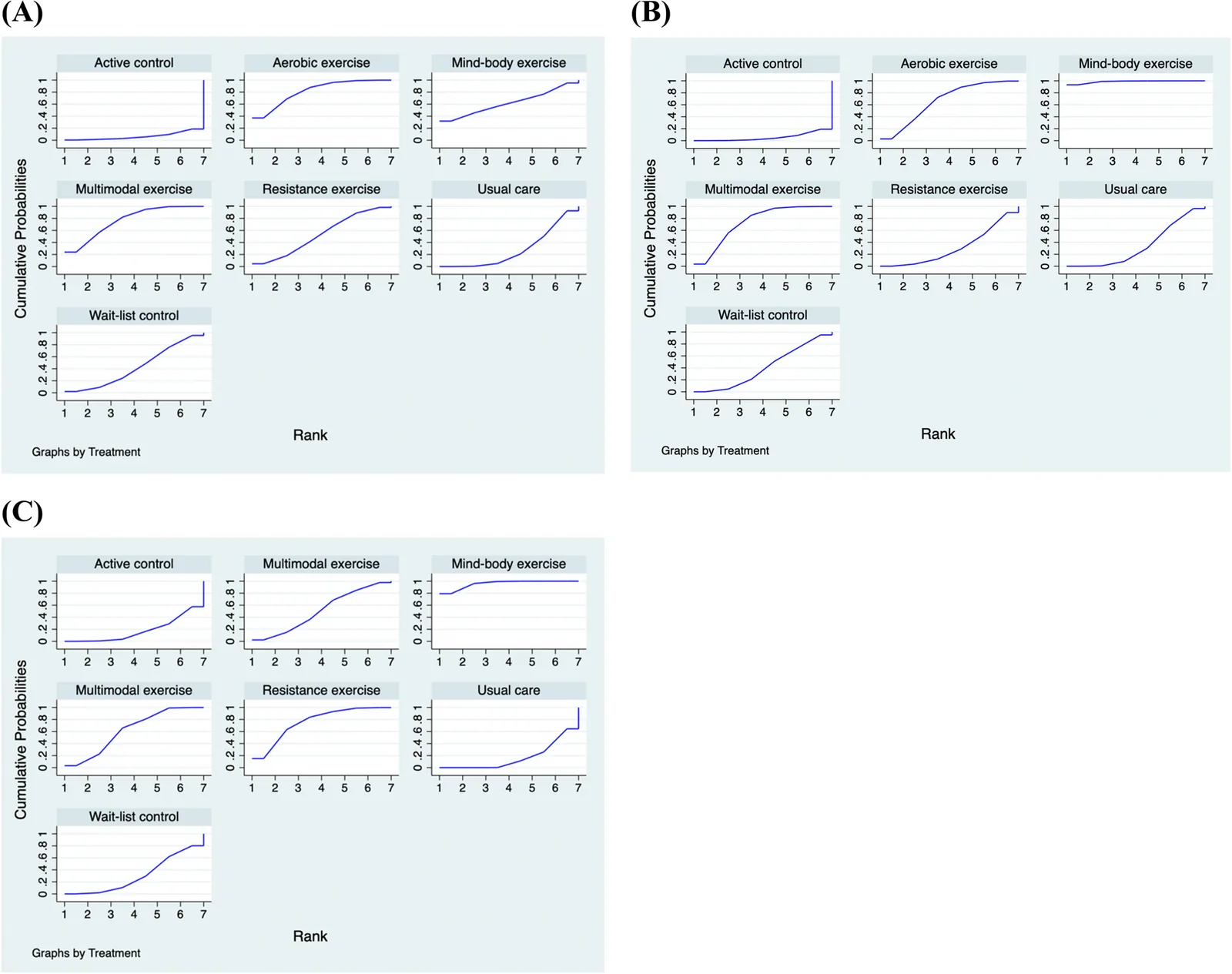
Cumulative probability ranking plots. (**A**) Quality of life, (**B**) Depression, (**C**) Anxiety

Based on SUCRA hierarchy (Table 3; Fig. 5A), AE achieved the highest SUCRA value (81.5%) and therefore ranked first in terms of ranking probability within this network, followed by ME (76.3%), MBE (62.0%), and RE (53.1%).

#### Depression

Fifteen studies examined the effects of different exercise types on improving depressive symptoms in older adults with cancer, involving 7 interventions. NMA showed statistically significant results as follows: MBE was significantly superior to usual care (SMD = -0.75, 95% CI: -1.22 to -0.28), waitlist control (SMD = -0.70, 95% CI: -1.20 to -0.19), and active control (SMD = -1.14, 95% CI: -1.67 to -0.61); MBE (SMD = -0.79, 95% CI: -1.31 to -0.26) was significantly superior to RE; ME (SMD = -0.70, 95% CI: -1.22 to -0.18) was significantly superior to active control. Beyond these significant results, most other comparisons showed positive trends, but their 95% CIs crossed zero (Fig. 4B, Figure S3.2).

Based on SUCRA hierarchy (Table 3; Fig. 5B), MBE achieved the highest SUCRA value (98.6%) and therefore ranked first in terms of ranking probability within this network, followed by ME (73.5%), AE (66.3%), and RE (31.2%).

#### Anxiety

Eleven studies examined the effects of different exercise types on improving anxiety symptoms in older adults with cancer, involving 7 interventions. NMA showed statistically significant results as follows: MBE (SMD = -0.70, 95% CI: -1.09 to -0.30), ME (SMD = -0.35, 95% CI: -0.54 to -0.16), and RE (SMD = -0.49, 95% CI: -0.88 to -0.10) were all significantly superior to usual care; MBE was significantly superior to active control (SMD = -0.70, 95% CI: -1.17 to -0.24) and waitlist control (SMD = -0.55, 95% CI: -1.02 to -0.08). Beyond these significant results, most other comparisons showed positive trends, but their 95% CIs crossed zero (Fig. 4C, Figure S3.3).

Based on SUCRA hierarchy (Table 3; Fig. 5C), MBE achieved the highest SUCRA value (95.8%) and therefore ranked first in terms of ranking probability within this network, followed by RE (75.7%), ME (62.0%), and AE (50.9%).

The NMA results for each outcome are summarized in league tables (Appendix 3), presenting SMD and 95% CI for all pairwise comparisons. Values at the intersection of rows and columns indicate the effect size for the comparison between the row and column interventions. When interpreting these results, the source of evidence (direct vs. indirect) should be considered; comparisons based solely on indirect evidence are less reliable and should be interpreted with caution.

**Table 3:** SUCRA ranking results

Intervention	Quality of life		Depression		Anxiety	
	SUCRA (%)	Mean Rank	SUCRA (%)	Mean Rank	SUCRA (%)	Mean Rank
Active control	6.4	6.6	5.6	6.7	17.9	5.9
Aerobic exercise	81.5	2.1	66.3	3.0	50.9	3.9
Mind-body exercise	62.0	3.3	98.6	1.1	95.8	1.3
Multimodal exercise	76.3	2.4	73.5	2.6	62.0	3.3
Resistance exercise	53.1	3.8	31.2	5.1	75.7	2.5
Usual Care	28.2	5.3	33.9	5.0	17.0	6.0
Waitlist Control	42.5	4.5	40.8	4.6	30.7	5.2

*SUCRA* Surface Under the Cumulative Ranking curve

### Heterogeneity and consistency analysis

Global consistency testing and the node-splitting method were used to assess model consistency and heterogeneity. Based on the magnitude of heterogeneity among studies, the effect model was selected. The results showed that the fixed-effect model was used for quality of life (*I²* = 42.0%) and anxiety (*I²* = 28.9%), while the random-effects model was used for depression (*I²* = 51.5%). Global consistency test results showed no significant global inconsistency for quality of life (*P* = 0.285), depression (*P* = 0.896), or anxiety (*P* = 0.310). Node-splitting analysis showed no significant differences between direct and indirect evidence for any comparison (*P* > 0.05). These results indicate good network consistency. However, non-significant inconsistency tests do not prove the absence of inconsistency, especially when direct evidence is limited, and statistical power is low. Thus, comparisons relying mainly on indirect evidence should be interpreted with caution.

### Subgroup analysis

The subgroup analysis results showed that the overall direction of effects of exercise interventions on quality of life, depression, and anxiety in older adults with cancer was generally consistent across subgroups defined by risk of bias, intervention duration, comparator type, cancer type, metastatic status, and disease phase. No between-subgroup differences sufficient to alter the overall conclusions were observed. There was some variation in effect sizes across different subgroups, suggesting that the above clinical factors may have a certain impact on intervention effects, but the overall findings remained stable. Due to the limited number of studies in some subgroups, the relevant results should be interpreted with caution. Detailed results are presented in Appendix 4.

### Publication bias

**Fig. 6:**
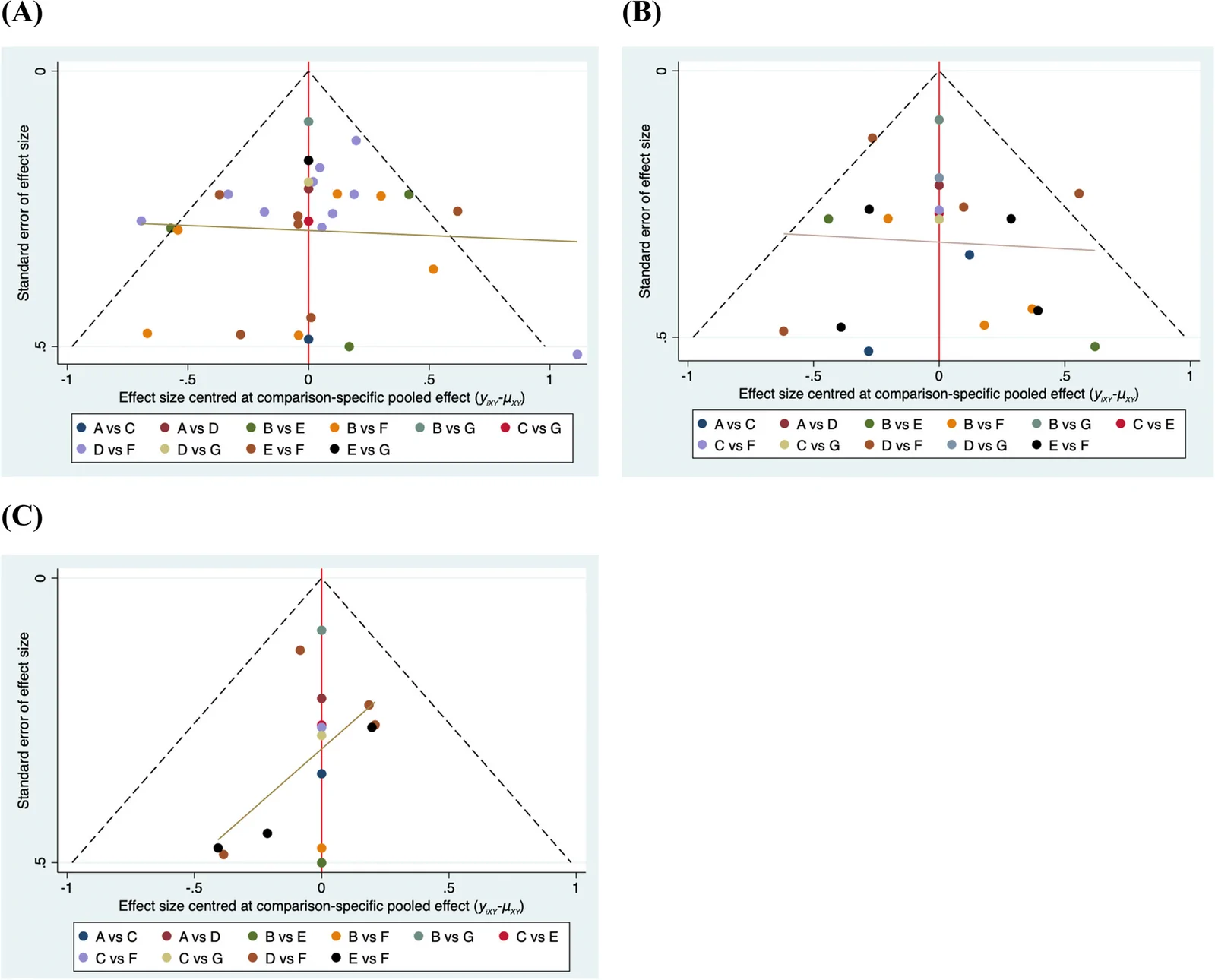
Comparison-adjusted funnel plots. (**A**) Quality of life, (**B**) Depression, (**C**) Anxiety

Publication bias was assessed using Egger’s test and funnel plots. Egger’s test results showed no significant publication bias for the effects of exercise interventions on quality of life (*P* = 0.160), depression (*P* = 0.511), or anxiety (*P* = 0.163), as all *P* values were greater than 0.05. Funnel plots showed that effect sizes from individual studies were generally symmetrically distributed around the pooled effect size (Fig. 6), further supporting the conclusion of no significant publication bias. These results indicate that the meta-analysis findings are robust. However, Egger’s test has limited power with a small number of studies, and this result cannot exclude publication bias. Therefore, it should be interpreted alongside sensitivity analyses.

### Sensitivity analysis

To test the robustness of the pooled effect sizes and assess the influence of individual studies, leave-one-out sensitivity analyses were conducted for quality of life, depression and anxiety outcomes. The results showed that the recalculated pooled effect sizes fluctuated minimally, and all fell within the 95% CIs of the original overall models (Appendix 5). This indicates that no single study had a substantial impact on the summary results, further confirming the reliability of the effect size estimates in this study. To assess the impact of model choice on the robustness of the results, we further estimated the quality-of-life outcome (*I²* = 42.0%) using a random-effects model. The results showed that the direction of effects was consistent across both models, with no substantial differences in effect sizes. This indicates that the choice of the fixed-effect model did not bias the conclusions, and the results are robust.

### Transitivity assessment

The boxplot distributions of age, sample size, intervention duration, and exercise frequency across intervention nodes are presented in Appendix 6. Regarding age, the median age of the four nodes ranged from approximately 68 to 72 years, with an overall range of approximately 65 to 74 years, indicating a highly comparable distribution. Regarding sample size, the median of each node ranged from 50 to 100 cases, but considerable variation existed across studies, with ME showing the greatest variability. Regarding intervention duration, AE, RE, and MBE had similar distributions, while ME showed slightly greater variability. Regarding exercise frequency, AE was mostly performed 3 times per week, RE 2–3 times per week, ME 2–5 times per week, and MBE 1–3 times per week; the distributions were generally comparable across nodes.

Prostate cancer predominated across all nodes, with ME covering a broader range of cancer types. Treatment phase was mainly radiotherapy or hormone therapy; MBE was mostly post-treatment, while ME covered all phases. Only three studies involved metastatic patients. Active treatment studies accounted for the majority, with survivorship/follow-up ≈ 10. Most studies were fully supervised, and usual care was the most common comparator. Outcome instruments were generally balanced across nodes. Exercise intensity standards varied, but all nodes targeted moderate intensity. Baseline psychological status could not be uniformly quantified due to diverse instruments (7 quality of life scales, 5 depressions, 4 anxiety); however, scores were generally at moderate (quality of life) or normal-to-mild (depression/anxiety) levels, with no systematic differences across nodes. Overall, transitivity was generally reasonable, but ME showed considerable variability in sample size, duration, and combinations, and metastatic/survivorship studies were limited, warranting cautious interpretation.

### Certainty of evidence assessment

The CINeMA assessment results are presented in Appendix 7. Most comparisons between exercise types and usual care were rated as moderate certainty; comparisons involving active controls or those entirely based on indirect evidence were rated as low to very low certainty. Overall, the certainty of evidence was limited, and all comparison results should be interpreted with caution.

## Discussion

This systematic review and NMA comprehensively evaluated the effects of AE, RE, MBE, and ME on quality of life, depressive symptoms, and anxiety symptoms in older adults with cancer. The results demonstrate differential effects of various exercise types on specific psychological outcomes, a finding that provides important evidence for individualized exercise prescription in this population.

### Overall effects of exercise interventions on psychological outcomes

This study found that various exercise interventions all had varying degrees of positive effects on psychological outcomes in older adults with cancer. This conclusion aligns with findings from previous systematic reviews. Soong et al. (2025) conducted a meta-analysis including 27 RCTs (1,929 participants) and similarly found that exercise interventions significantly reduced depression and anxiety levels and improved health-related quality of life in older adults with cancer [11]. Subgroup analyses in that study further revealed that MBE (e.g., Tai Chi, Yoga, Qigong) were more effective for depression and anxiety than traditional exercise forms, highly consistent with our finding that MBE ranked best for depression and anxiety [11]. A systematic review by Farì et al. (2025) also confirmed that exercise interventions, including AE, RE, and MBE, positively improve depressive and anxiety symptoms in cancer patients, demonstrating good feasibility and effectiveness across different cancer types [49]. That study noted that the prevalence of depression and anxiety in cancer patients is approximately 20% to 25%, and these psychological issues not only reduce quality of life but may also affect treatment adherence and survival prognosis [49].

From a mechanistic perspective, the biological basis for exercise-induced improvement in psychological outcomes in older adults with cancer involves complex interactions across multiple levels. First, regular exercise can modulate the functional activity of the hypothalamic-pituitary-adrenal axis, reducing baseline levels of stress hormones such as cortisol, thereby mitigating the negative impact of chronic stress on mental health [50]. Second, exercise-induced muscle contraction promotes the release of myokines from peripheral tissues; these factors not only participate in energy metabolism regulation but can also cross the blood-brain barrier to influence the expression of neurotrophic factors in the central nervous system [51]. Additionally, exercise-mediated improvement in systemic low-grade inflammation, reflected by decreased levels of pro-inflammatory cytokines, contributes to alleviating inflammation-related affective symptoms [52]. For older adults with cancer, these physiological effects may be particularly important due to age-related neuroendocrine function changes and inflammation [53].

SUCRA rankings reflect probability rankings within a specific network, not definitive evidence of superiority [54]. Some comparisons lacked direct evidence and relied on indirect estimates with considerable uncertainty. Substantial heterogeneity existed within each exercise type—AE included walking, treadmill, and cycling; MBE involved yoga, tai chi, and qigong. Thus, mechanistic explanations should be viewed as inferences from current evidence, not definitive conclusions, and clinical application requires individualized judgment. Transitivity is a prerequisite for NMA reliability. Age and baseline psychological symptoms were generally balanced across exercise categories, supporting transitivity. However, prostate cancer predominated across all nodes, and ME duration varied considerably. Cancer type imbalance and intervention duration heterogeneity may affect transitivity. Therefore, generalization to non-prostate cancer populations should be cautious, and comparisons involving ME should be interpreted prudently.

### Preferential effect of mind-body exercise on depression and anxiety

A key finding of this study is that MBE ranked best for improving depressive and anxiety symptoms in older adults with cancer. This result also showed good efficacy in improving quality of life, suggesting that MBE may exert its psychological regulatory effects through unique mechanistic pathways. MBE (including Yoga, Tai Chi, Qigong, etc.) are characterized by integrating physical activity with cognitive regulation, respiratory rhythm, and interoceptive awareness. A systematic review by Corrêa et al. (2025) noted that multimodal intervention strategies, including MBE, positively impact the physical and mental health and quality of life of older adults with cancer [55]. That study included 15 RCTs and found that individual exercise, group exercise, home-based exercise, and ME programs all produced positive effects on physical and psychological health in older adults with cancer [55]. The meta-analysis by Soong et al. further confirmed that MBE was superior to conventional exercise forms for improving both depression and anxiety [11]. The authors noted that this was the first meta-analysis comparing the effects of MBE versus traditional exercise on psychological outcomes specifically in older adults with cancer, providing strong evidence supporting the preferential recommendation of MBE [11].

MBE may enhance anti-anxiety and antidepressant effects through the following mechanisms: First, the unique mindfulness meditation component of MBE can enhance patients’ awareness of negative emotions and a non-judgmental accepting attitude, thereby reducing emotional reactivity and rumination frequency [56]. Second, rhythmic breathing and slow movements can activate the parasympathetic nervous system, directly lowering physiological arousal levels and anxiety experience [57]. Third, the low-intensity nature of MBE makes it easier for older patients with limited physical reserve to adhere to, reducing psychological resistance caused by excessively high exercise intensity [58]. Furthermore, a RCT by Campo et al. (2014) demonstrated that a 12-week Qigong intervention significantly reduced psychological distress and fatigue in prostate cancer survivors, further supporting the applicability and effectiveness of MBE in the older adult cancer population [31]. A RCT by Cramer et al. (2016) similarly found that a 10-week Yoga intervention significantly improved anxiety and depressive symptoms in colorectal cancer patients, with more pronounced effects in the older adult subgroup [36].

Among the four MBE RCTs included yoga [35, 36], qigong [31], and tai chi [44] all improved depressive and anxiety symptoms. Yoga significantly improved both outcomes in two studies [35, 36], suggesting it may be the best-supported MBE type. However, due to heterogeneity in cancer type, intervention duration, and comparators, and the lack of head-to-head RCTs, no definitive conclusion can be drawn on which subtype is superior. Future head-to-head RCTs are needed. Based on the included studies and the needs of older adults with cancer, we propose a preliminary framework: yoga, tai chi, or qigong (yoga has relatively stronger evidence); 1–3 sessions/week; ~60 min/session; at least 8–12 weeks; low-to-moderate intensity. However, given the heterogeneity and lack of dose-response research, these recommendations should be viewed as exploratory. Future RCTs are needed to evaluate dose-response relationships and establish evidence-based prescriptions.

### Effects of aerobic and multimodal exercise on quality-of-life improvement

For improving quality of life, AE and ME showed superior effects in this study. This finding aligns with the recommendations of the American College of Sports Medicine (ACSM) exercise guidelines for cancer survivors, which recommend regular AE and RE to improve physical function and quality of life [6]. The systematic review by Corrêa et al. noted that ME programs, which combine physical exercise with stress reduction strategies, nutritional guidance, and monitoring, show positive evidence for improving rehabilitation and overall function in older adults with cancer [55]. That study found that individual exercise interventions (including AE and RE prescriptions) improved cardiorespiratory fitness, lower limb strength, and functional capacity; ME strategies improved quality of life, physical function, symptom burden, and depressive and anxiety symptoms [55].

Oppong et al. (2026) conducted a systematic review evaluating the effects of AE and RE across eight cancer types, including 25 RCTs [59]. Results showed that of the 13 trials reporting quality of life, 12 observed improvements; of the 10 studies reporting cancer-related fatigue, nine showed reduction [59]. The study also found that moderate-intensity, thrice-weekly exercise programs showed the most consistent benefits [59]. These findings provide evidence supporting the widespread implementation of AE and RE in cancer rehabilitation. A meta-analysis by Mustian et al. (2017) comparing the effects of pharmacological, psychological, and exercise interventions on cancer-related fatigue found that exercise interventions (including AE and MBE) had the best effect-risk ratio for improving fatigue [60].

The improvement in quality of life from AE is likely achieved primarily by enhancing cardiorespiratory fitness, reducing cancer-related fatigue, and improving physical activity capacity [60]. Older adults with cancer often experience diminished physiological reserve and limited activity ability, and AE can specifically target these functional deficits, thereby improving activities of daily living and health-related quality of life. A RCT by Segal et al. (2009) confirmed that a 24-week AE intervention significantly improved quality of life in prostate cancer patients, with particularly pronounced effects in older patients [24]. The advantage of ME lies in integrating the synergistic effects of multiple exercise forms, simultaneously producing comprehensive improvements across multiple health outcomes. A RCT by Cormie et al. (2015) showed that ME, including both aerobic and resistance components, simultaneously improved physical function, fatigue, and quality of life in prostate cancer patients [33]. A systematic review and meta-analysis by Sweegers et al. (2018) further confirmed that the effect of exercise interventions on improving health-related quality of life in cancer survivors was most significant for combined exercise modalities (AE plus RE) [61]. Although the direct effects of RE on psychological outcomes are relatively limited, it may indirectly produce psychological benefits by improving physical function and self-efficacy. A RCT by Winters-Stone et al. (2016) confirmed that a 6-month RE program significantly improved muscle strength, physical function, and quality of life in older breast cancer survivors [62]. A recently published NMA similarly comparing the effects of different exercise types on cancer survivors showed that AE ranked first for improving health-related quality of life (SUCRA = 92.0%), MBE was most effective for alleviating cancer-related fatigue, and ME performed best for improving physical function [13]. Further studies have indicated that RE combined with AE can significantly improve quality of life, body composition, and physical fitness in prostate cancer patients, but evidence for AE alone is insufficient [63].

The 27 RCTs included in this study covered a variety of cancer types, including prostate, lung, breast, colorectal, pancreatic, head and neck, and bladder cancers. However, prostate cancer predominated across all exercise nodes, with limited studies on other cancer types, which may limit the generalizability of the findings to non-prostate cancer populations. Additionally, multiple instruments were used to measure the same outcomes; although the use of SMD partially mitigated scale differences, variations in measurement precision and content focus across instruments may still introduce measurement heterogeneity. These factors may affect the reliability of indirect comparisons and treatment rankings, suggesting that the observed effect trends should be extrapolated with caution across different cancer types and measurement contexts.

### Clinical practice implications and exercise prescription recommendations

Based on the findings of this study and considering existing evidence, exercise prescriptions for older adults with cancer should follow an individualized and symptom-oriented approach. For older adults with cancer whose primary concerns are depression or anxiety, MBE (yoga, tai chi, qigong) may be considered a promising exercise option. For patients whose core goal is improving quality of life, AE and ME are better choices. However, a scoping review by Winters-Stone et al. (2025) noted that the research evidence currently available to guide exercise prescriptions for older adults with cancer remains limited [58]. This review screened 1,790 articles and ultimately identified only six controlled trials meeting inclusion criteria. The study found trial recruitment rates ranged from 21% to 37%, adherence rates from 48% to 84%, and retention rates from 61% to 77% [58]. These data indicate that conducting exercise intervention studies in older adults with cancer faces unique challenges, including recruitment difficulties, fluctuating adherence, and suboptimal retention. A systematic review by Geidl et al. (2020) further explored the dose-response relationship between physical activity and mortality, finding that even an increase of 10 metabolic equivalent hours of physical activity per week in breast cancer survivors was associated with a 22% reduction in mortality, emphasizing the importance of promoting physical activity in cancer survivors, but also noting that precise exercise prescription parameters for patients with non-communicable diseases remain to be clarified [64].

It is noteworthy that older adults with cancer often have multiple comorbidities, cognitive decline, and weakened social support, factors that may affect the implementation effectiveness and adherence to exercise interventions [53, 65]. A review by Loh et al. (2018) emphasized that exercise interventions can effectively improve biopsychosocial function, including mental health issues such as depression and anxiety, in older adults with cancer. However, their implementation in clinical practice faces multiple challenges, including determining the optimal exercise dose for specific outcomes, assessing exercise risks and safety, and effectively promoting exercise programs at individual, healthcare team, and organizational levels [66]. Therefore, exercise prescription should comprehensively consider the patient’s physical function, cognitive status, social environment, and treatment phase, adopting phased, progressive implementation strategies [16]. Schmitz et al. (2019), in their “Exercise is Medicine” in oncology guideline, indicated that integrating exercise into routine cancer care requires multidisciplinary collaboration and individualized program design [67]. Home-based exercise programs, group exercise formats, and remote supervised guidance may help improve exercise participation and long-term adherence in older patients. A recent scoping review by Kim et al. indicated that physical activity interventions based on digital health interventions can effectively alleviate cancer-related fatigue in cancer survivors, with wearable devices and long-term interventions exceeding 12 weeks showing the most significant effects. However, existing evidence is primarily focused on breast cancer populations and lacks systematic integration [68]. A study by Loh et al. (2018) confirmed that a 6-week ME intervention significantly improved quality of life and psychological distress in older breast cancer patients, suggesting that short-term, structured exercise programs have good feasibility and effects in the older adult population [66]. Research by Galvão et al. (2018) further showed that even for prostate cancer patients with bone metastases, professionally supervised exercise remains safe and effective, significantly improving physical function and delaying disease-related functional decline [69]. Additionally, a RCT by Kleckner et al. explored the effects of exercise on symptoms during chemotherapy, finding that individualized, progressive home-based walking and RE programs effectively alleviated chemotherapy-induced peripheral neuropathy symptoms, suggesting a potential synergistic effect between exercise and conventional anticancer therapy, providing empirical evidence for exercise combined with pharmacotherapy in managing cancer symptoms [70].

Beyond the type of exercise, the mode of intervention delivery also influences the benefits older patients derive from exercise. Traditional center-based programs face practical barriers such as transportation and cost, which home-based telerehabilitation can effectively supplement. Large-scale population studies have confirmed that physical inactivity is associated with higher mortality risk in cancer patients, and structured home-based exercise interventions can effectively improve this situation [71]. Evidence indicates that telehealth-supported home-based exercise is safe and feasible in cancer survivors, significantly improving cardiorespiratory fitness and physical activity levels, with adherence comparable to center-based training and lower healthcare costs [11, 72–75]. A randomized controlled trial in lymphoma survivors further demonstrated that home-based exercise is non-inferior to center-based training in improving cardiorespiratory fitness and physical function, with lower costs [76]. A network meta-analysis comparing different exercise types also supported the improvement of quality of life through ME [77]. Therefore, future efforts should establish a tiered, accessible rehabilitation service network that integrates home-based telerehabilitation into the exercise prescription system for older adults with cancer, to overcome the accessibility limitations of traditional models.

### Strengths and limitations

The strengths of this study include its focus on older adults with cancer, the use of NMA to enable indirect comparisons and ranking of multiple exercise types, and the high quality of the included studies with manageable risk of bias. However, several limitations should be acknowledged. First, considerable clinical heterogeneity existed across the included studies, with inconsistent intervention protocols within each exercise type—AE included walking, stationary cycling, and treadmill training; RE varied in load settings and exercise selection; MBE involved different schools such as yoga, tai chi, and qigong; and ME differed in combination modalities and dose ratios—all of which may have affected the precision of effect estimates. Second, the limited number of direct comparisons between some exercise types resulted in wide confidence intervals for indirect comparisons; particularly, the insufficient number of studies on mind-body subtypes precluded determination of which form is most effective. Third, although sensitivity analyses did not identify undue influence from individual studies, the leave-one-out method cannot fully address uncertainties related to sparse networks and indirect evidence. Fourth, the inclusion criterion of a mean sample age ≥ 65 years may have allowed the inclusion of some participants < 65 years, potentially affecting generalizability to the older adult population. Fifth, most studies had short follow-up periods (≤ 6 months), leaving the long-term effects of exercise interventions unclear. Sixth, blinding of participants to exercise interventions was not feasible, potentially introducing performance and detection bias. Seventh, the restriction to English-language literature and the predominance of studies from high-income countries may introduce language bias and limit generalizability. Eighth, the three comparator types differed in levels of attention and activity exposure, with active control potentially underestimating the true effect of exercise interventions. Ninth, non-significant statistical consistency tests do not establish the plausibility of the transitivity assumption, as the two address different issues. Tenth, the limited statistical power of Egger’s test for depression (15 studies) and anxiety (11 studies) cannot completely rule out publication bias. Eleventh, baseline psychological symptom severity varied across studies, and although SMD can integrate different scale scores, it cannot adjust for the impact of baseline severity on absolute benefits, limiting generalizability to populations with different levels of symptom severity.

### Future research directions

Based on the above limitations, future research should explore the following six directions. First, head-to-head comparative trials among MBE subtypes should be conducted. Given that yoga, Tai Chi, and Qigong have all shown positive trends in improving depression and anxiety, yet direct comparative evidence among these subtypes remains lacking, future three-arm RCTs are warranted to compare the efficacy of the three MBE forms within the same population of older patients with cancer. Second, a standardized research framework for exercise dose-response relationships should be established. The current studies exhibit considerable heterogeneity in frequency, intensity, session duration, and total intervention length across different exercise types. Future research should adopt stepwise dose-escalation designs to systematically evaluate the dose-response curves of various exercise doses on psychological outcomes. Third, the coverage of cancer types should be expanded. Given that prostate cancer predominates across all exercise nodes in this study, future investigations should purposefully enroll older patients with other common cancers, such as lung, breast, and colorectal cancers, to test the generalizability of the findings. Fourth, follow-up periods should be extended. Most included studies only reported immediate post-intervention effects. Future studies should include follow-up assessments of at least six months or longer to evaluate the long-term maintenance of exercise-induced benefits. Fifth, the statistical power for subgroup analyses should be enhanced. The current subgroup analyses are constrained by limited study numbers. Future research should pre-specify adequately powered subgroup analyses to assess the modifying effects of metastatic status, treatment phase, and baseline psychological symptom severity on exercise outcomes. Sixth, core outcome measurement instruments should be standardized. It is recommended that future studies adopt internationally recognized core outcome sets, such as the EORTC QLQ-C30 for quality of life and the HADS or PHQ-9/GAD-7 for psychological symptoms, to improve between-study comparability and the quality of NMA.

## Conclusions

This systematic review and NMA compared the effects of four exercise types on quality of life, depression, and anxiety in older patients with cancer. The results indicated that different exercise types may exert differential effects on specific psychological outcomes. MBE, represented by yoga, Tai Chi, and Qigong, ranked highest for alleviating depression and anxiety, with yoga showing relatively more concentrated supportive evidence. AE and ME ranked higher for improving quality of life. However, these findings should be interpreted with caution given the clinical heterogeneity among included studies, the lack of direct evidence for some comparisons, and the low to moderate certainty of the evidence. Therefore, the ranking results should serve as preliminary reference rather than definitive recommendations. Future head-to-head RCTs directly comparing different mind-body exercise subtypes, along with dose-response studies to establish optimal exercise prescription parameters, are urgently needed. Extended follow-up periods and expanded coverage of cancer types are also warranted to establish more evidence-based precision exercise prescriptions.

## Supplementary Information

Supplementary Material 1.

## Data Availability

The datasets used and analyzed during the current study are available from the corresponding author on reasonable request.

## Abbreviations

NMA: Network Meta-Analysis
RCT: Randomized Controlled Trial
SMD: Standardized Mean Difference
CI: Confidence Interval
SUCRA: Surface Under the Cumulative Ranking Curve
RoB 2: Risk of Bias tool version 2.0
MBE: Mind-body exercise
AE: Aerobic exercise
RE: Resistance exercise
ME: Multimodal exercise
HADS: Hospital Anxiety and Depression Scale
PHQ-9: Patient Health Questionnaire-9
STAI: State-Trait Anxiety Inventory
