# Supplementary material for: Comparative efficacy of different exercise interventions for improving quality of life, depression, and anxiety in older adults with cancer: a systematic review and network meta-analysis
Source: BMC Geriatr. 2026 Jul 25;26:1161. doi: 10.1186/s12877-026-08022-4 (PMC13570530; doi:10.1186/s12877-026-08022-4)
Supplement: Supplementary file 1 [file 12877_2026_8022_MOESM1_ESM.docx]

Supplementary Material.

**Table of contents**

[**Appendix 1. PRISMA-NMA checklist**	2](#_Toc235022903)

[**Appendix 2. Search strategy**	6](#_Toc235022904)

[**Appendix 3. League table**	8](#_Toc235022905)

[**Appendix 4. Subgroup analysis**	9](#_Toc235022906)

[**Appendix 5. Sensitivity analysis**	18](#_Toc235022907)

[**Appendix 6. Boxplots**	20](#_Toc235022908)

[**Appendix 7. CINeMA Assessment**	22](#_Toc235022909)

# **Appendix 1. PRISMA-NMA checklist**

**Table S1.** PRISMA NMA Checklist of Items to Include When Reporting A Systematic Review Involving a Network Meta-analysis.

| **Section/Topic** | **Item #** | **Checklist Item** | **Reported on Page #** |
| --- | --- | --- | --- |
| **TITLE** |  |  |  |
| Title | 1 | Identify the report as a systematic review *incorporating a network meta-analysis (or related form of meta-analysis).* | **Page 1** |
|  |  |  |  |
| **ABSTRACT** |  |  |  |
| Structured summary | 2 | Provide a structured summary including, as applicable:  **Background:** main objectives  **Methods:** data sources; study eligibility criteria, participants, and interventions; study appraisal; and *synthesis methods, such as network meta-analysis.*  **Results:** number of studies and participants identified; summary estimates with corresponding confidence/credible intervals; *treatment rankings may also be discussed. Authors may choose to summarize pairwise comparisons against a chosen treatment included in their analyses for brevity.*  **Discussion/Conclusions:** limitations; conclusions and implications of findings.  **Other:** primary source of funding; systematic review registration number with registry name. | **Page 2-3** |
|  |  |  |  |
| **INTRODUCTION** |  |  |  |
| Rationale | 3 | Describe the rationale for the review in the context of what is already known*, including mention of why a network meta-analysis has been conducted.* | **Page 3-5** |
| Objectives | 4 | Provide an explicit statement of questions being addressed, with reference to participants, interventions, comparisons, outcomes, and study design (PICOS). | **Page 5-7** |
|  |  |  |  |
| **METHODS** |  |  |  |
| Protocol and registration | 5 | Indicate whether a review protocol exists and if and where it can be accessed (e.g., Web address); and, if available, provide registration information, including registration number. | **Page 7** |
| Eligibility criteria | 6 | Specify study characteristics (e.g., PICOS, length of follow-up) and report characteristics (e.g., years considered, language, publication status) used as criteria for eligibility, giving rationale. *Clearly describe eligible treatments included in the treatment network, and note whether any have been clustered or merged into the same node (with justification).* | **Page 8-10** |
| Information sources | 7 | Describe all information sources (e.g., databases with dates of coverage, contact with study authors to identify additional studies) in the search and date last searched. | **Page 7-8** |
| Search | 8 | Present full electronic search strategy for at least one database, including any limits used, such that it could be repeated. | **Page 7-8** |
| Study selection | 9 | State the process for selecting studies (i.e., screening, eligibility, included in systematic review, and, if applicable, included in the meta-analysis). | **Page 10** |
| Data collection process | 10 | Describe method of data extraction from reports (e.g., piloted forms, independently, in duplicate) and any processes for obtaining and confirming data from investigators. | **Page 10** |
| Data items | 11 | List and define all variables for which data were sought (e.g., PICOS, funding sources) and any assumptions and simplifications made. | **Page 8-11** |
| **Geometry of the network** | **S1** | Describe methods used to explore the geometry of the treatment network under study and potential biases related to it. This should include how the evidence base has been graphically summarized for presentation, and what characteristics were compiled and used to describe the evidence base to readers. | **Page 12** |
| Risk of bias within individual studies | 12 | Describe methods used for assessing risk of bias of individual studies (including specification of whether this was done at the study or outcome level), and how this information is to be used in any data synthesis. | **Page 11** |
| Summary measures | 13 | State the principal summary measures (e.g., risk ratio, difference in means). *Also describe the use of additional summary measures assessed, such as treatment rankings and surface under the cumulative ranking curve (SUCRA) values, as well as modified approaches used to present summary findings from meta-analyses.* | **Page 12-13** |
| Planned methods of analysis | 14 | Describe the methods of handling data and combining results of studies for each network meta-analysis. This should include, but not be limited to:   - *Handling of multi-arm trials;* - *Selection of variance structure;* - *Selection of prior distributions in Bayesian analyses; and* - *Assessment of model fit.* | **Page 13** |
| **Assessment of Inconsistency** | **S2** | Describe the statistical methods used to evaluate the agreement of direct and indirect evidence in the treatment network(s) studied. Describe efforts taken to address its presence when found. | **Page 13** |
| Risk of bias across studies | 15 | Specify any assessment of risk of bias that may affect the cumulative evidence (e.g., publication bias, selective reporting within studies). | **Page 14** |
| Additional analyses | 16 | Describe methods of additional analyses if done, indicating which were pre-specified. This may include, but not be limited to, the following:   - Sensitivity or subgroup analyses; - Meta-regression analyses; - *Alternative formulations of the treatment network; and* - *Use of alternative prior distributions for Bayesian analyses (if applicable).* | **Page 13-15** |
|  |  |  |  |
| **RESULTS†** |  |  |  |
| Study selection | 17 | Give numbers of studies screened, assessed for eligibility, and included in the review, with reasons for exclusions at each stage, ideally with a flow diagram. | **Page 15** |
| **Presentation of network structure** | **S3** | Provide a network graph of the included studies to enable visualization of the geometry of the treatment network. | **Page 27-29** |
| **Summary of network geometry** | **S4** | Provide a brief overview of characteristics of the treatment network. This may include commentary on the abundance of trials and randomized patients for the different interventions and pairwise comparisons in the network, gaps of evidence in the treatment network, and potential biases reflected by the network structure. | **Page 28** |
| Study characteristics | 18 | For each study, present characteristics for which data were extracted (e.g., study size, PICOS, follow-up period) and provide the citations. | **Page 16-25** |
| Risk of bias within studies | 19 | Present data on risk of bias of each study and, if available, any outcome level assessment. | **Page 26-27** |
| Results of individual studies | 20 | For all outcomes considered (benefits or harms), present, for each study: 1) simple summary data for each intervention group, and 2) effect estimates and confidence intervals. *Modified approaches may be needed to deal with information from larger networks.* | **Page 29-33** |
| Synthesis of results | 21 | Present results of each meta-analysis done, including confidence/credible intervals. *In larger networks, authors may focus on comparisons versus a particular comparator (e.g. placebo or standard care), with full findings presented in an appendix. League tables and forest plots may be considered to summarize pairwise comparisons.* If additional summary measures were explored (such as treatment rankings), these should also be presented. | **Page 29-34** |
| **Exploration for inconsistency** | **S5** | Describe results from investigations of inconsistency. This may include such information as measures of model fit to compare consistency and inconsistency models, *P* values from statistical tests, or summary of inconsistency estimates from different parts of the treatment network. | **Page 34-35** |
| Risk of bias across studies | 22 | Present results of any assessment of risk of bias across studies for the evidence base being studied. | **Page 35-37** |
| Results of additional analyses | 23 | Give results of additional analyses, if done (e.g., sensitivity or subgroup analyses, meta-regression analyses*, alternative network geometries studied, alternative choice of prior distributions for Bayesian analyses,* and so forth). | **Page 37-39** |
|  |  |  |  |
| **DISCUSSION** |  |  |  |
| Summary of evidence | 24 | Summarize the main findings, including the strength of evidence for each main outcome; consider their relevance to key groups (e.g., healthcare providers, users, and policy-makers). | **Page 39-50** |
| Limitations | 25 | Discuss limitations at study and outcome level (e.g., risk of bias), and at review level (e.g., incomplete retrieval of identified research, reporting bias). *Comment on the validity of the assumptions, such as transitivity and consistency. Comment on any concerns regarding network geometry (e.g., avoidance of certain comparisons).* | **Page 50-51** |
| Conclusions | 26 | Provide a general interpretation of the results in the context of other evidence, and implications for future research. | **Page 51** |
|  |  |  |  |
| **FUNDING** |  |  |  |
| Funding | 27 | Describe sources of funding for the systematic review and other support (e.g., supply of data); role of funders for the systematic review. This should also include information regarding whether funding has been received from manufacturers of treatments in the network and/or whether some of the authors are content experts with professional conflicts of interest that could affect use of treatments in the network. | **Page 53** |

PICOS = population, intervention, comparators, outcomes, study design.

* Text in italics indicateS wording specific to reporting of network meta-analyses that has been added to guidance from the PRISMA statement.

† Authors may wish to plan for use of appendices to present all relevant information in full detail for items in this section.

# **Appendix 2. Search strategy**

**Table S2.** Database search strategy.

| **Database** | Search strategies |
| --- | --- |
| **PubMed** | **[#1]** (((((((((((((((((((((("Resistance Training"[Mesh]) OR ("resistance training"[Title/Abstract])) OR ("strength training"[Title/Abstract])) OR ("aerobic exercise"[Title/Abstract])) OR ("aerobic training"[Title/Abstract])) OR ("physical activity"[Title/Abstract])) OR ("physical exercise"[Title/Abstract])) OR ("exercise"[Title/Abstract])) OR ("yoga"[Title/Abstract])) OR ("tai chi"[Title/Abstract])) OR ("tai ji"[Title/Abstract])) OR ("qigong"[Title/Abstract])) OR ("mind-body exercise"[Title/Abstract])) OR ("multimodal exercise"[Title/Abstract])) OR ("combined exercise"[Title/Abstract])) OR ("walking"[Title/Abstract])) OR ("jogging"[Title/Abstract])) OR ("running"[Title/Abstract])) OR ("cycling"[Title/Abstract])) OR ("swimming"[Title/Abstract]))  **[#2]** (((((((((((((("Aged"[Mesh]) OR ("Geriatrics"[Mesh])) OR ("older adult"[Title/Abstract])) OR ("elderly"[Title/Abstract])) OR ("aged"[Title/Abstract])) OR ("geriatric"[Title/Abstract])) OR ("geriatrics"[Title/Abstract])) OR ("older people"[Title/Abstract])) OR ("older person"[Title/Abstract])) OR ("older patient"[Title/Abstract])) OR ("senior"[Title/Abstract])) OR ("aging"[Title/Abstract])) OR ("ageing"[Title/Abstract]))  **[#3]**(((((((((((((("Neoplasms"[Mesh]) OR ("cancer"[Title/Abstract])) OR ("tumor"[Title/Abstract])) OR ("tumour"[Title/Abstract])) OR ("malignancy"[Title/Abstract])) OR ("malignancies"[Title/Abstract])) OR ("carcinoma"[Title/Abstract])) OR ("sarcoma"[Title/Abstract])) OR ("leukemia"[Title/Abstract])) OR ("lymphoma"[Title/Abstract])) OR ("neoplasm"[Title/Abstract])) OR ("oncology"[Title/Abstract]))  **[#4]** (((((((((((((((("Depression"[Mesh]) OR ("Anxiety"[Mesh])) OR ("Quality of Life"[Mesh])) OR ("depression"[Title/Abstract])) OR ("depressive symptom"[Title/Abstract])) OR ("anxiety"[Title/Abstract])) OR ("anxious"[Title/Abstract])) OR ("quality of life"[Title/Abstract])) OR ("HRQOL"[Title/Abstract])) OR ("health-related quality of life"[Title/Abstract])) OR ("mental health"[Title/Abstract])) OR ("psychological distress"[Title/Abstract])) OR ("mood"[Title/Abstract])) OR ("emotional well-being"[Title/Abstract]))  **[#5]**  (((((((((((((("Randomized Controlled Trial"[Publication Type]) OR ("randomized"[Title/Abstract])) OR ("randomised"[Title/Abstract])) OR ("RCT"[Title/Abstract])) OR ("randomly"[Title/Abstract])) OR ("controlled clinical trial"[Title/Abstract])) OR ("clinical trial"[Title/Abstract])) OR ("placebo"[Title/Abstract])) OR ("trial"[Title/Abstract])) OR ("random allocation"[Title/Abstract]))  **[#6] #1 AND #2 AND #3 AND #4 AND #5** |
| **Embase** | **[#1]** ('resistance training'/exp OR 'strength training'/exp OR 'aerobic exercise'/exp OR 'aerobic training'/exp OR 'physical activity'/exp OR 'physical exercise'/exp OR 'exercise'/exp OR 'yoga'/exp OR 'tai chi'/exp OR 'qigong'/exp OR 'mind-body exercise'/exp OR 'multimodal exercise'/exp OR 'combined exercise'/exp OR 'walking'/exp OR 'jogging'/exp OR 'running'/exp OR 'cycling'/exp OR 'swimming'/exp):ab,kw,ti  **[#2]** ('aged'/exp OR 'geriatrics'/exp OR 'older adult':ab,kw,ti OR 'older adults':ab,kw,ti OR 'elderly':ab,kw,ti OR 'aged':ab,kw,ti OR 'geriatric':ab,kw,ti OR 'geriatrics':ab,kw,ti OR 'older people':ab,kw,ti OR 'older person':ab,kw,ti OR 'senior':ab,kw,ti OR 'seniors':ab,kw,ti OR 'aging':ab,kw,ti OR 'ageing':ab,kw,ti)  **[#3]** ('neoplasm'/exp OR 'cancer'/exp OR 'cancer':ab,kw,ti OR 'tumor':ab,kw,ti OR 'tumour':ab,kw,ti OR 'malignancy':ab,kw,ti OR 'malignancies':ab,kw,ti OR 'carcinoma':ab,kw,ti OR 'sarcoma':ab,kw,ti OR 'leukemia':ab,kw,ti OR 'lymphoma':ab,kw,ti OR 'neoplasm':ab,kw,ti OR 'oncology':ab,kw,ti)  **[#4]** ('depression'/exp OR 'anxiety'/exp OR 'quality of life'/exp OR 'depression':ab,kw,ti OR 'depressive symptom':ab,kw,ti OR 'anxiety':ab,kw,ti OR 'anxious':ab,kw,ti OR 'quality of life':ab,kw,ti OR 'HRQOL':ab,kw,ti OR 'health-related quality of life':ab,kw,ti OR 'mental health':ab,kw,ti OR 'psychological distress':ab,kw,ti OR 'mood':ab,kw,ti OR 'emotional well-being':ab,kw,ti)  **[#5]** ('randomized controlled trial'/exp OR 'clinical trial'/exp OR 'randomized controlled trial':ab,kw,ti OR 'RCT':ab,kw,ti OR 'randomized':ab,kw,ti OR 'randomised':ab,kw,ti OR 'randomly':ab,kw,ti OR 'placebo':ab,kw,ti OR 'clinical trial':ab,kw,ti)  **[#6] #1 AND #2 AND #3 AND #4 AND #5** |
| **Cochrane library** | **[#1]**（MeSH descriptor: [Resistance Training] explode all trees OR MeSH descriptor: [Exercise] explode all trees OR (resistance training OR strength training OR aerobic exercise OR aerobic training OR physical activity OR physical exercise OR exercise OR yoga OR tai chi OR qigong OR mind-body exercise OR multimodal exercise OR combined exercise OR walking OR jogging OR running OR cycling OR swimming):ti,ab,kw）  **[#2]** (MeSH descriptor: [Aged] explode all trees OR MeSH descriptor: [Geriatrics] explode all trees OR "older adult":ti,ab,kw OR "older adults":ti,ab,kw OR "elderly":ti,ab,kw OR "aged":ti,ab,kw OR "geriatric":ti,ab,kw OR "geriatrics":ti,ab,kw OR "older people":ti,ab,kw OR "older person":ti,ab,kw OR "senior":ti,ab,kw OR "seniors":ti,ab,kw OR "aging":ti,ab,kw OR "ageing":ti,ab,kw)  **[#3]** (MeSH descriptor: [Neoplasms] explode all trees OR "cancer":ti,ab,kw OR "tumor":ti,ab,kw OR "tumour":ti,ab,kw OR "malignancy":ti,ab,kw OR "malignancies":ti,ab,kw OR "carcinoma":ti,ab,kw OR "sarcoma":ti,ab,kw OR "leukemia":ti,ab,kw OR "lymphoma":ti,ab,kw OR "neoplasm":ti,ab,kw OR "oncology":ti,ab,kw)  **[#4]** (MeSH descriptor: [Depression] explode all trees OR MeSH descriptor: [Anxiety] explode all trees OR MeSH descriptor: [Quality of Life] explode all trees OR "depression":ti,ab,kw OR "depressive symptom":ti,ab,kw OR "anxiety":ti,ab,kw OR "anxious":ti,ab,kw OR "quality of life":ti,ab,kw OR "HRQOL":ti,ab,kw OR "health-related quality of life":ti,ab,kw OR "mental health":ti,ab,kw OR "psychological distress":ti,ab,kw OR "mood":ti,ab,kw OR "emotional well-being":ti,ab,kw)  **[#5]** (MeSH descriptor: [Randomized Controlled Trial] explode all trees OR "randomized controlled trial":ti,ab,kw OR "RCT":ti,ab,kw OR "randomized":ti,ab,kw OR "randomised":ti,ab,kw OR "randomly":ti,ab,kw OR "placebo":ti,ab,kw OR "clinical trial":ti,ab,kw)  **[#6] #1 AND #2 AND #3 AND #4 AND #5** |
| **Web of Science** | **[#1]** TS=("Resistance training" OR "strength training" OR "aerobic exercise" OR "aerobic training" OR "physical activity" OR "physical exercise" OR "exercise" OR "yoga" OR "tai chi" OR "qigong" OR "mind-body exercise" OR "multimodal exercise" OR "combined exercise" OR "walking" OR "jogging" OR "running" OR "cycling" OR "swimming")  **[#2]** TS=("Aged" OR "Geriatrics" OR "older adult*" OR "elderly" OR "aged" OR "geriatric" OR "geriatrics" OR "older people" OR "older person*" OR "older patient*" OR "senior*" OR "aging" OR "ageing")  **[#3]** TS=(Neoplasms OR cancer OR tumor OR tumour OR malignancy OR malignancies OR carcinoma OR sarcoma OR leukemia OR lymphoma OR neoplasm OR oncology)  **[#4]** TS=(Depression OR Anxiety OR "Quality of Life" OR depression OR "depressive symptom*" OR anxiety OR anxious OR "quality of life" OR HRQOL OR "health-related quality of life" OR "mental health" OR "psychological distress" OR mood OR "emotional well-being")  **[#5]** T TS=("Randomized Controlled Trial" OR randomized OR randomised OR RCT OR randomly OR "controlled clinical trial" OR "clinical trial" OR placebo OR trial OR "random allocation")  **#6] #1 AND #2 AND #3 AND #4 AND #5** |

# **Appendix 3. League table**

**Table S3.1.** League table for network comparisons of quality of life (SMD, 95% CI).

**Table S3.2.** League table for network comparisons of depression (SMD, 95% CI).

**Table S3.3.** League table for network comparisons of anxiety (SMD, 95% CI).

# **Appendix 4. Subgroup analysis**

**Figure S4.1.** Forest plot of subgroup analysis for quality of life by risk of bias.

**Figure S4.2.** Forest plot of subgroup analysis for quality of life by duration.

**Figure S4.3.** Forest plot of subgroup analysis for quality of life by control group.

**Figure S4.4.** Forest plot of subgroup analysis for quality of life by cancer type.

**Figure S4.5.** Forest plot of subgroup analysis for quality of life by metastatic status.

**Figure S4.6.** Forest plot of subgroup analysis for quality of life by survivorship phase.

**Figure S4.7.** Forest plot of subgroup analysis for depression by risk of bias.

**Figure S4.8.** Forest plot of subgroup analysis for depression by duration.

**Figure S4.9.** Forest plot of subgroup analysis for depression by control group.

**Figure S4.10.** Forest plot of subgroup analysis for depression by cancer type.

**Figure S4.11.** Forest plot of subgroup analysis for depression by metastatic status.

**Figure S4.12.** Forest plot of subgroup analysis for depression by survivorship phase.

**Figure S4.13.** Forest plot of subgroup analysis for anxiety by risk of bias.

**Figure S4.14.** Forest plot of subgroup analysis for anxiety by duration.

**Figure S4.15.** Forest plot of subgroup analysis for anxiety by control group.

**Figure S4.16.** Forest plot of subgroup analysis for anxiety by cancer type.

**Figure S4.17.** Forest plot of subgroup analysis for anxiety by metastatic status.

**Figure S4.18.** Forest plot of subgroup analysis for anxiety by survivorship phase.

# **Appendix 5. Sensitivity analysis**

**Figure S5.1.** Sensitivity analysis plot of the effects of exercise interventions on quality of life in older adults with cancer.

**Figure S5.2.** Sensitivity analysis plot of the effects of exercise interventions on depressive symptoms in older adults with cancer.

**Figure S5.3.** Sensitivity analysis plot of the effects of exercise interventions on anxiety symptoms in older adults with cancer.

**Figure S5.4.** Forest plot of quality of life using random-effects model.

# **Appendix 6. Boxplots**

**Figure S6.1.** Boxplots of age as an effect modifier across intervention nodes.

**Figure S6.2.** Boxplots of sample as an effect modifier across intervention nodes.

**Figure S6.3.** Boxplots of duration as an effect modifier across intervention nodes.

**Figure S6.4.** Boxplots of exercise frequency as an effect modifier across intervention nodes.

# **Appendix 7. CINeMA Assessment**

**Figure S7.1.** Risk of bias contribution by intervention group in quality of life.

**Figure S7.2.** Overall risk of bias by treatment comparison in quality of life.

**Table S7.1.** CINeMA results of quality of life.

| Comparison | Number of studies | Within-  study bias | Reporting bias | Indirectness | Imprecision | Heterogeneity | Incoherence | Confidence rating |
| --- | --- | --- | --- | --- | --- | --- | --- | --- |
| AC:MBE | 1 | Some concerns | Low risk | No concerns | Some concerns | No concerns | No concerns | Low |
| AC:ME | 1 | Major concerns | Low risk | No concerns | No concerns | Some concerns | No concerns | Very low |
| AE:RE | 3 | Some concerns | Low risk | No concerns | No concerns | No concerns | No concerns | Moderate |
| AE:UC | 6 | Some concerns | Low risk | No concerns | No concerns | Some concerns | No concerns | Low |
| AE:WLC | 1 | No concerns | Low risk | No concerns | No concerns | No concerns | No concerns | High |
| MBE:WLC | 1 | Major concerns | Low risk | No concerns | No concerns | Some concerns | No concerns | Very low |
| ME:UC | 10 | Some concerns | Low risk | No concerns | No concerns | No concerns | No concerns | Moderate |
| ME:WL | 1 | Major concerns | Low risk | No concerns | No concerns | Some concerns | Major concerns | Very low |
| RE:UC | 6 | Some concerns | Low risk | No concerns | No concerns | No concerns | No concerns | Moderate |
| RE:WLC | 1 | Some concerns | Low risk | No concerns | No concerns | No concerns | No concerns | Moderate |
| AE, aerobic exercise; MBE, mind-body exercise; ME, multimodal exercise; RE, resistance exercise; WLC, waitlist control; UC, usual care; AC, active control. | | | | | | | | |

**Figure S7.3.** Risk of bias contribution by intervention group in depression.

**Figure S7.4.** Overall risk of bias by treatment comparison in depression.

**Table S7.2.** CINeMA results of depression.

| Comparison | Number of studies | Within-  study bias | Reporting bias | Indirectness | Imprecision | Heterogeneity | Incoherence | Confidence rating |
| --- | --- | --- | --- | --- | --- | --- | --- | --- |
| AC:MBE | 2 | Major concerns | Low risk | No concerns | No concerns | No concerns | No concerns | Low |
| AC:ME | 1 | Major concerns | Low risk | No concerns | No concerns | Some concerns | No concerns | Very low |
| AE:RE | 2 | Some concerns | Low risk | No concerns | No concerns | Some concerns | No concerns | Low |
| AE:UC | 3 | Some concerns | Low risk | No concerns | No concerns | No concerns | No concerns | Moderate |
| AE:WLC | 1 | No concerns | Low risk | No concerns | No concerns | No concerns | No concerns | High |
| MBE:RE | 1 | Some concerns | Low risk | No concerns | No concerns | Some concerns | No concerns | Low |
| MBE:UC | 1 | Some concerns | Low risk | No concerns | No concerns | No concerns | No concerns | Moderate |
| MBE:WLC | 1 | Major concerns | Low risk | No concerns | No concerns | Some concerns | No concerns | Very low |
| ME:UC | 4 | Some concerns | Low risk | No concerns | No concerns | No concerns | No concerns | Moderate |
| ME:WLC | 1 | Major concerns | Low risk | No concerns | No concerns | No concerns | No concerns | Low |
| RE:UC | 4 | Some concerns | Low risk | No concerns | No concerns | No concerns | No concerns | Moderate |
| AE, aerobic exercise; MBE, mind-body exercise; ME, multimodal exercise; RE, resistance exercise; WLC, waitlist control; UC, usual care; AC, active control. | | | | | | | | |

**Figure S7.5.** Risk of bias contribution by intervention group in anxiety.

**Figure S7.6.** Overall risk of bias by treatment comparison in anxiety.

**Table S7.3.** CINeMA results of anxiety.

| Comparison | Number of studies | Within-  study bias | Reporting bias | Indirectness | Imprecision | Heterogeneity | Incoherence | Confidence rating |
| --- | --- | --- | --- | --- | --- | --- | --- | --- |
| AC:MBE | 1 | Major concerns | Low risk | No concerns | No concerns | No concerns | Some concerns | Very low |
| AC:ME | 1 | Major concerns | Low risk | No concerns | No concerns | No concerns | Some concerns | Very low |
| AE:RE | 1 | Some concerns | Low risk | No concerns | No concerns | No concerns | No concerns | Moderate |
| AE:UC | 1 | Some concerns | Low risk | No concerns | No concerns | No concerns | No concerns | Moderate |
| AE:WLC | 1 | No concerns | Low risk | No concerns | No concerns | No concerns | No concerns | High |
| MBE:RE | 1 | Some concerns | Low risk | No concerns | No concerns | No concerns | No concerns | Moderate |
| MBE:UC | 1 | Some concerns | Low risk | No concerns | No concerns | No concerns | No concerns | Moderate |
| MBE:WLC | 1 | Major concerns | Low risk | No concerns | No concerns | Some concerns | No concerns | Very low |
| ME:UC | 4 | Major concerns | Low risk | No concerns | No concerns | No concerns | Some concerns | Very low |
| RE:UC | 3 | Some concerns | Low risk | No concerns | No concerns | No concerns | Some concerns | Low |
| AE, aerobic exercise; MBE, mind-body exercise; ME, multimodal exercise; RE, resistance exercise; WLC, waitlist control; UC, usual care; AC, active control. | | | | | | | | |
